# Human neonatal MR1T cells have more diverse TCR repertoires but reduced bacterial recognition than adult MR1T cells

**DOI:** 10.1038/s41467-026-74998-7

**Published:** 2026-06-29

**Authors:** Dylan Kain, Wael Awad, G. W. McElfresh, Meghan Cansler, Gwendolyn M. Swarbrick, Kean Chan Yew Poa, Conor McNeice, Gregory Boggy, Katherine H. Rott, Megan D. Null, David M. Lewinsohn, Jamie Rossjohn, Benjamin N. Bimber, Deborah A. Lewinsohn

**Affiliations:** 1 Division of Infectious Diseases, Department of Pediatrics, Oregon Health & Science University, Portland, OR USA; 2 Division of Pulmonary, Allergy and Critical Care Medicine, Department of Medicine, Oregon Health & Science University, Portland, OR USA; 3 Division of Infectious Diseases, Department of Medicine, University of Toronto, Toronto, ON Canada; 4 Infection and Immunity Program and Department of Biochemistry and Molecular Biology, Biomedicine Discovery Institute, Monash University, Clayton, VIC Australia; 5 Oregon National Primate Research Center, OHSU, Beaverton, OR USA; 6 Institute of Infection and Immunity, Cardiff University, School of Medicine, Heath Park, Cardiff, UK; 7 Vaccine and Gene Therapy Institute, OHSU, Beaverton, OR USA

**Keywords:** Neonatal sepsis, T cells, Systems analysis

## Abstract

Bacterial sepsis is a leading cause of neonatal mortality. Pro-inflammatory MR1-restricted T (MR1T) cells may help protect from sepsis by recognizing bacterial pathogens producing the canonical MR1 antigen 5-OP-RU. Most adult MR1T cells are mucosal-associated invariant T (MAIT) cells expressing a semi-invariant TCRα, while neonatal MR1T cells express diverse TCRα chains. Here, we perform combined single-cell RNA-sequencing and TCR repertoire analyses on MR1/5-OP-RU tetramer-positive cells from neonatal cord blood (CB) and adult blood. Compared to adult MR1T cells, CB MR1T cells exhibit greater TCR diversity, reduced cytotoxic and proinflammatory gene expression, diminished bacterial recognition and reduced binding to MR1/5-OP-RU. Structural analysis of a CB MAIT TCR reveals decreased β chain contribution to the TCR–MR1 interface relative to an adult MAIT TCR. These findings demonstrate developmental stage-specific differences in MR1T cell repertoire, function and MAIT TCR structure with implications for neonatal sepsis.

## Abstract (web summary)

MR1T cells may contribute to protection from neonatal sepsis, but comparisons between adult and neonatal MR1T cells are scarce. Here, the authors use cord blood and adult blood MR1T cells for scRNA-seq and TCR-seq, revealing increased TCR diversity, yet poor recognition of pathogens by neonatal MR1T cells.

## Introduction

Neonatal sepsis is one of the leading causes of death in children, accounting for an estimated 400,000–700,000 deaths annually^1^. Tuberculosis (TB), caused by *M. tuberculosis* (Mtb), is another leading cause of death in children, accounting for nearly a quarter million deaths a year^2^. Neonates are particularly vulnerable to infections, including Mtb, in part due to a naïve and underdeveloped immune system. Understanding the immune system response in early life, and the way in which different immune cells develop can inform vaccine strategies to provide protection in early life from a variety of infections, including TB.

MR1-restricted T (MR1T) cells are defined by their recognition of small molecules including microbial^3–7^, self ^8,9^, and drug and drug-like molecules^10^ presented by the monomorphic MHC class 1-related molecule (MR1). In adults, the majority of MR1T cells, known as mucosal associated invariant T (MAIT) cells, express an invariant T cell receptor (TCR) α-chain (TRAV1-2/TRAJ33/20/12 in humans), paired with a limited array of Vβ segments^11^, and express high levels of CD161^12,13^ and CD26^13^. MR1T cell populations which express TCRα-chains other than TRAV1-2 have been described^6,14^. Here, we define MAIT cells as a subset of MR1T cells that are TRAV1-2(+) and MR1T cells that express TCRα-chains other than TRAV1-2 as TRAV 1-2 (-) MR1T cells. Riboflavin metabolites are dominant MR1 ligands^7,15^, and thus MAIT cells recognize riboflavin-producing organisms^3,4^ and have been shown in mouse models to be important for immune defense against many common childhood pathogens including *Klebsiella pneumoniae*^16^*, E. coli*^4^, and *S. pneumoniae*^17^. TRAV1-2(-) MR1T cells can respond to a broader array of microbial ligands, including recognition of microorganisms that cannot produce riboflavin^6^. TRAV1-2(-) MR1T cells also recognize non-microbial ligands and may play additional roles in cancer immunology, auto-immunity and other immune driven diseases^7,9,18,19^.

TRAV1-2(-) MR1T cells represent the majority of MR1T cells in cord blood (CB) samples^20^. By contrast, while a TRAV1-2(-) MR1T cell population persists in adults, MAIT cells represent the vast majority of MR1T cells. By 10-weeks of age, however, infant MAIT cells are present in similar frequencies as in adult participants^20^. Furthermore, infant MAIT cells, were able to produce TNF in response to *Mycobacterium smegmatis* at a similar level to adult MAIT cells, while in CB there were fewer MAIT cells that produced TNF^20^. The rapid shift from abundant TRAV1-2(-) MR1T cells at birth, to more functional MAIT cells by 10-weeks of age, suggests rapid expansion of MAIT cells early in life, possibly driven by microbial exposures, such as those from the microbiota^21^.

It remains unclear whether the predominance of naïve MR1T cells in cord blood contributes to heightened susceptibility to bacterial pathogens in early life. Here, we combine single-cell transcriptomic and TCR repertoire analyses to define the developmental and functional properties of neonatal MR1T cells relative to adults. Cord blood MAIT and TRAV1-2(–) MR1T cells display reduced pro-inflammatory and cytotoxic gene programs together with increased TCR diversity. Clonal analyses demonstrate that these features persist at the single-clone level and associate with impaired recognition of common bacterial pathogens. Biochemical and structural analyses further reveal diminished binding to the canonical MR1 ligand 5-OP-RU and altered β-chain contribution at the TCR–MR1 interface. Collectively, these findings define intrinsic developmental differences in neonatal MR1T cells that limit antimicrobial function. These results provide mechanistic insight into early-life vulnerability to bacterial infection and may inform strategies aimed at enhancing MR1T-mediated immunity in newborns.

## Results

### CB MAIT and TRAV1-2(-) MR1T cells are less cytotoxic and pro-inflammatory

**Fig. 1:**
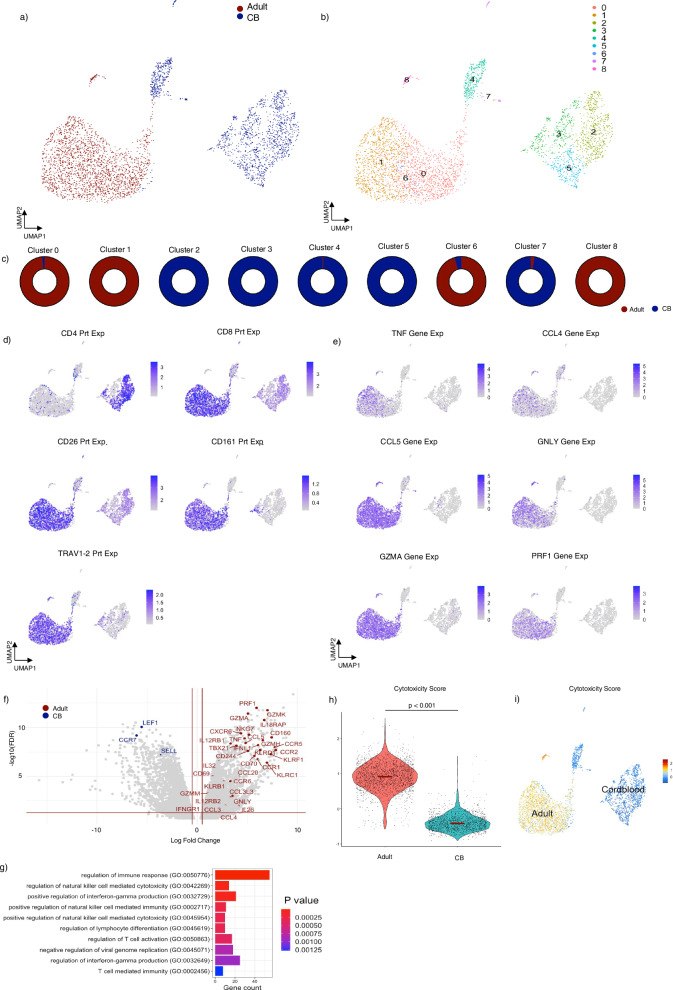
Cord blood MR1T cells have a distinct, less cytotoxic gene expression profile compared to adult MR1T cells. **a** Dimensionality reduction of all MR1/5-OP-RU sorted MR1-restricted T (MR1T) cells from 5 adult donors and 5 cord blood (CB) donors (*n* = 2200 adult MR1T cells and 1,513 CB MR1T cells). **b** Unsupervised clustering of all MR1T cells. **c** Percent of each cluster that is made up by each donor type (adult vs CB). **d** Cell surface protein expression by CITE-seq staining. Prt Exp = Protein expression. **e** Gene expression of common inflammatory/cytotoxic genes. Gene Exp = Gene expression. **f** Volcano plot of differentially expressed genes in CB vs adult MR1T cells. *p*-values were calculated using the Wilcoxon signed-rank test with Bonferroni adjustment for multiple comparisons. Horizontal red line represents Bonferroni-adjusted *p*-value of 0.05 and vertical red line represents log fold change of −0.5 or 0.5. **g** Top 10 upregulated gene ontology pathways in adult MR1T cells compared to CB MR1T cells. *p*-values were calculated using the Wilcoxon signed-rank test with Bonferroni adjustment for multiple comparisons. **h**, **i** Cytotoxicity score (*PRF1, GNLY, NKG7, GZMA, GZMB, GZMH, GZMK* and *GZMM*) for adult compared to CB MR1T cells. *p* < 0.0001 using the Wilcoxon signed-rank test.

To investigate the phenotype and function of MR1T cells in CB, we performed scGEX-seq/scTCR-seq on MR1/5-OP-RU tetramer(+) cells from CB (*n* = 5) as well as adult controls (*n* = 5). The experiment included 5 oligo-conjugated antibodies for cell surface protein expression at single-cell resolution. After quality control and filtering steps, there were 2200 adult and 1,513 CB MR1T cells included in the analysis. CB-derived MR1T cells were transcriptionally distinct from adult MR1T cells (Fig. 1a). Unsupervised clustering resulted in 9 distinct clusters (Fig. 1b) with each cluster predominantly made up of cells from either adult or CB participants (Fig. 1c). Cell surface protein expression was different between CB and adult participants, as adult cells were predominantly MAIT cells, expressing not only TRAV1-2 but also CD8, CD26, and CD161, while CB cells were predominantly TRAV1-2, CD161, and CD26 negative and more likely to express CD4 (Fig. 1d). In CB, CD4^+^ T cells clustered separately from CD8^+^ T cells, whereas in adults, rare CD4^+^ T cells were dispersed throughout the UMAP. Adult MR1T cells also showed increased expression of pro-inflammatory and cytotoxic genes, (*TNF, CCL4, GNLY, GZMA, PRF1*), indicating a more effector-differentiated phenotype (Fig. 1e).

Differential gene expression between CB and adult MR1T cells yielded 3083 upregulated genes in adults and 3157 upregulated genes in CB cells (Fig. 1f, Supplemental Data 1). Notably, adult cells differentially expressed many pro-inflammatory and cytotoxic genes (*PRF1, GZMA, GZMH, GZMM, GZMK, GNLY, CCL3, CCL4, CCR5, CCR2, CCR1, TNF, NKG7, IL32, IL26*), meanwhile, CB cells had increased expression of naïve markers (*LEF1, CCR7, SELL*). Top upregulated pathways in adult MR1T cells included several pro-inflammatory and activation pathways (Fig. 1g). To illustrate these differences, we scored cells for enrichment of a gene module associated with cytotoxic differentiation, comprised of *PRF1, GNLY, NKG7, GZMA, GZMB, GMZK, GZMM*, using the UCell package^22^. Unstimulated adult cells were more cytotoxic than CB MR1T cells (Fig. 1h, i). In CB participants the majority of TRAV1-2(-) cells were CD4-positive and CD8-negative, whereas in adult participants the majority of cells were CD8-positive irrespective of TRAV1-2 status (Supplemental Fig. 1).

### CB MR1T cells demonstrate far more TCR repertoire diversity

**Fig. 2:**
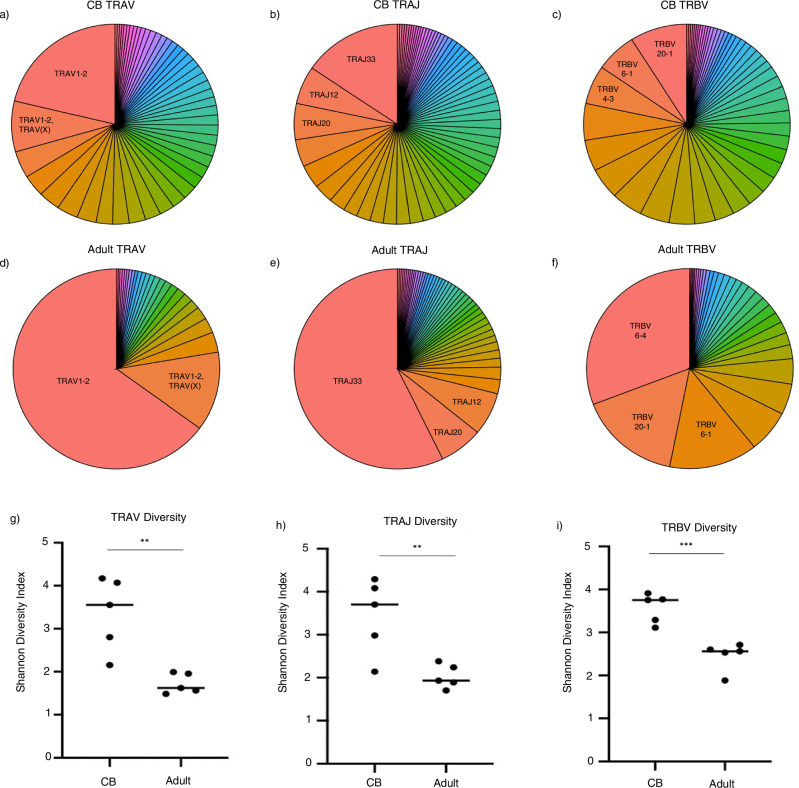
Cord blood MR1T cells demonstrate a much more diverse TCR repertoire compared to adult MR1T cells. **a**–**f** Cord blood (CB) and adult MR1-restricted T (MR1T) cell TRAV, TRAJ and TRBV repertoire by TCR sequencing. TRAV1-2,TRAV(X) represents cells with dual expression of alpha chains, one being TRAV1-2 and TRAV(X) representing any TRAV1-2(-). **g**–**i** Shannon diversity index of TRAV, TRAJ and TRBV repertoire of CB and adult MR1T cells. Unpaired two-tailed *t*-test with *p*-value < 0.01 denoted by ** and *p*-value < 0.001 denoted by ***. *n* = 5 per group. TRAV *p*-value = 0.0036; TRAJ *p* = 0.0091; TRBV *p* = 0.0008.

Next, we examined the diversity of TCR usage in adult and CB MR1T cells, finding significantly more diversity in CB samples (Fig. 2). Surface TRAV1-2 staining was much lower for CB cells (Fig. 1d) and this was confirmed through scTCR-seq, demonstrating that TRAV1-2 represents less than a quarter of all TCRs used (Fig. 2a). Conversely, while the vast majority of adult MR1T cells express TRAV1-2, a population of MR1T cells expressing other TRAV genes are present in adults (Fig. 2d). Furthermore, while canonical TRAJ chains, TRAJ33/20/12, are also the most common TRAJ chains used by CB cells, they represent a far smaller subset of total TRAJ compared to adult cells (Fig. 2b, e). CB TRBV chains also showed more diversity compared to adult MR1T cells (Fig. 2c, f). This diversity is quantified using the Shannon diversity index, showing significantly more diversity in CB TCRs (Fig. 2g–i). Furthermore, adult participants were found to have more expanded clonotypes, with 19.1% (107/560) of cells having at least one other cell with an identical CDR3, whereas only 0.1% (1/959) of CB cells were expanded clonotypes (Supplemental Fig. 2).

**Fig. 3:**
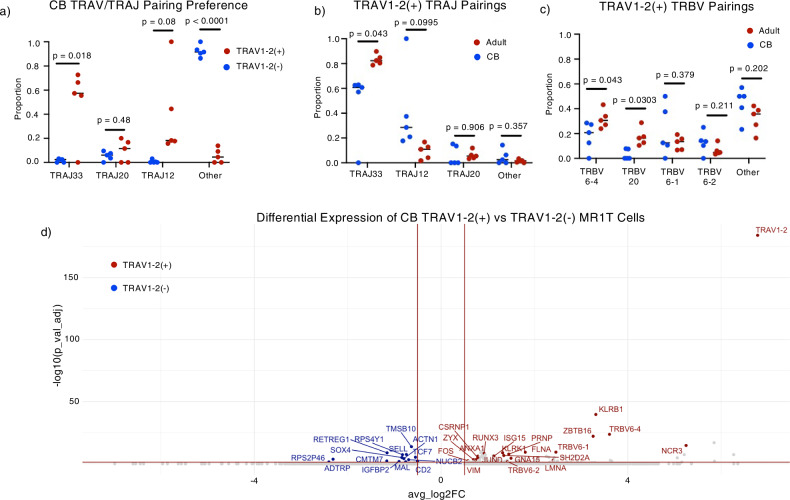
Pairing preferences are more diverse for CB MR1T cells. **a** Pairing preferences of cord blood (CB) MR1-restricted T (MR1T) cells based on gene expression of TRAV1-2(+) vs TRAV1-2(-). Unpaired two-sided t-test. *n* = 5 per group. **b** TRAJ pairing preference for TRAV1-2( + ) MR1T cells from adult and CB donors. Unpaired *t*-test. **c** TRBV pairing preference for TRAV1-2( + ) MR1T cells from adult and CB donors. Unpaired t-test. **d** Volcano plot for differential gene expression of CB MR1T cells expressing TRAV1-2(+) or TRAV1-2(-). *p*-values were calculated using the Wilcoxon signed-rank test with Bonferroni adjustment for multiple comparisons. Labeled genes were greater than 0.5 or less than -0.5 log fold change and had a Bonferroni-adjusted *p*-value of <0.05.

Previous work has shown that dual TCR-alpha expression can confound MAIT cell TCR interpretation^23^. To confirm that this would not be the driving force in TCR interpretation, we examined cell surface protein expression of TRAV1-2 utilizing CITE-seq staining in CB participants, comparing cells with TRAV1-2 gene expression compared to those without (Supplemental Fig. 3). TRAV1-2 gene expressing cells had much higher cell surface expression of TRAV1-2, suggesting dual TCR-alpha expression would not significantly affect our subsequent analysis. Given the high frequency of TRAV1-2(-) MR1T cells in the CB participants, we examined if TRAV usage would impact TRAJ or TRBV pairings. Utilizing TCR sequences from CB participants, we compared TRAJ usage for TRAV1-2( + ) MAIT cells compared to TRAV1-2(-) MR1T cells (Fig. 3a). TRAV1-2(+) cells had significantly higher pairing with TRAJ33, whereas TRAV1-2(-) cells had significantly higher pairing preference with non-canonical TRAJs (non-TRAJ33/20/12). This suggests TRAV1-2 usage influences TRAJ usage in MR1T cells. We next explored if there were pairing preference differences in MAIT cells from CB compared to adult participants. Examining only TRAV1-2(+) cells, we compared TRAJ usage between adult and CB participants (Fig. 3b). Even among MAIT cells, adult participants had significantly higher canonical TRAJ33 pairings compared to CB participants. Adult participants’ MAIT cells also had significantly higher pairing preference for TRBV20-1 (Fig. 3c).

### Unstimulated CB MAIT cells are more mature than CB TRAV1-2(-) MR1T cells

Given the increased gene expression of pro-inflammatory and cytotoxic genes in adult-derived MR1T cells, and the much higher percentage of MAIT cells in adults, we hypothesized that MAIT cells in CB would also have higher expression of pro-inflammatory and cytotoxic gene expression. Therefore, we examined subsets of CB-derived MAIT and TRAV1-2(-) MR1T cells and performed differential gene expression between TRAV1-2(+) and TRAV1-2(-) cell populations. This yielded 15 genes that were significantly upregulated in TRAV1-2(-) cells and 80 genes that were significantly upregulated in TRAV1-2-(+) cells (Fig. 3d and Supplemental Data 2). As expected, *TRAV1-2* was the most increased gene among TRAV1-2(+) cells. TRBV genes, *TRBV6-1/TRBV6-4*, were also significantly upregulated in TRAV1-2(+) cells, as they were more likely to express TRBV6-1, TRBV6-2 or TRBV6-4. Other genes that were significantly upregulated in TRAV1-2(+) cells include effector function genes (*KLRB1, KLRK1, NCR3, ZBTB16*)^12^, genes associated with T-cell-activation (*LMNA, ANXA1, PRNP, CSNP1*)^24–26^, genes involved in cell proliferation and cell signaling (*SH2D2D, JUND, FOS, GNA15*)^27–29^, interferon associated genes (*ISG15, ZYX*)^30^ and genes involved in tissue residency or tissue trafficking (*RUNX3, VIM, FLNA*)^24,31,32^. Although there are limited significantly upregulated genes in TRAV1-2(-) cells, notably they do upregulate *SELL*, (encoding for CD62L/L-selectin), whose expression is generally high on naïve T cells^33^.

### 5-OP-RU stimulation preferentially expands MAIT cells compared to TRAV1-2(-) MR1T cells in vitro in cord blood

**Fig. 4:**
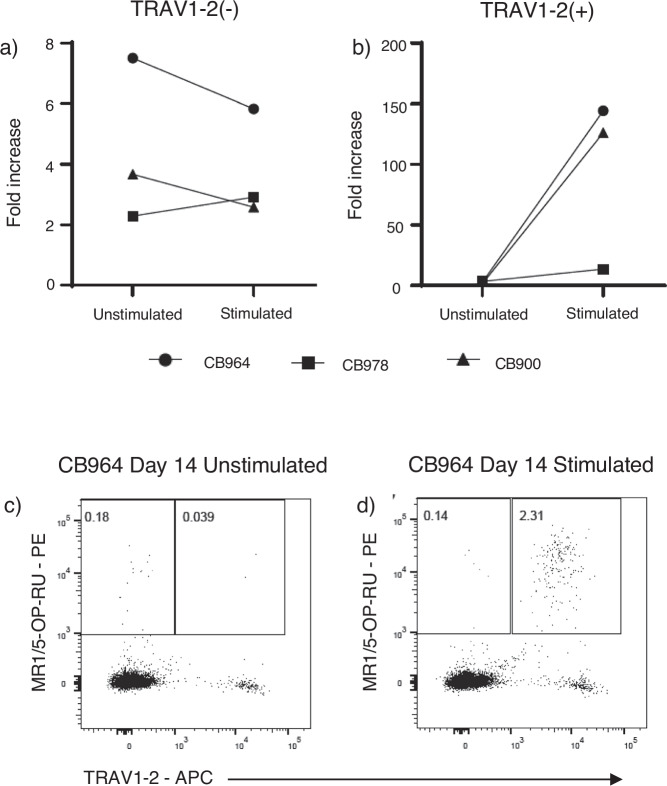
5-OP-RU preferentially expands MAIT cells compared to TRAV1-2(-) MR1T cells. **a**–**b** Cord blood mononuclear cells (CBMC; *n* = 3) had flow phenotyping performed on day 0. The remaining cells were either stimulated on day 1 and day 7 with 5-OP-RU (1 µM) or unstimulated. Cells were harvested and analyzed for flow cytometry on Day 14. Cells were gated on Live, CD3 + MR1/5-OP-RU+ and either TRAV1-2(+) or TRAV1-2(−). The fold increase on day 14 compared to the day 0 frequencies is shown for both the unstimulated and 5-OP-RU stimulated MR1-restricted T (MR1T) cells. **c**–**d** Representative flow plots of CB964 on day 14 unstimulated or 5-OP-RU stimulated.

Given the upregulation of effector function, T cell activation, and cell proliferation genes in CB MAIT cells as compared to CB TRAV1-2(-) MRIT cells, we hypothesized that 5-OP-RU would selectively expand MAIT cells in CBMC samples when exposed to 5-OP-RU in vitro. To address this hypothesis, CBMC (*n* = 3) were stimulated with 5-OP-RU over a 14-day period (Fig. 4a–d, Supplemental Fig. 4). Stimulation with 5-OP-RU resulted in dramatic expansion of MAIT cells. By comparison, TRAV1-2(-) MR1T cells persisted in culture but did not expand.

### Adult MR1T cells are more cytotoxic than CB MR1T cells

**Fig. 5:**
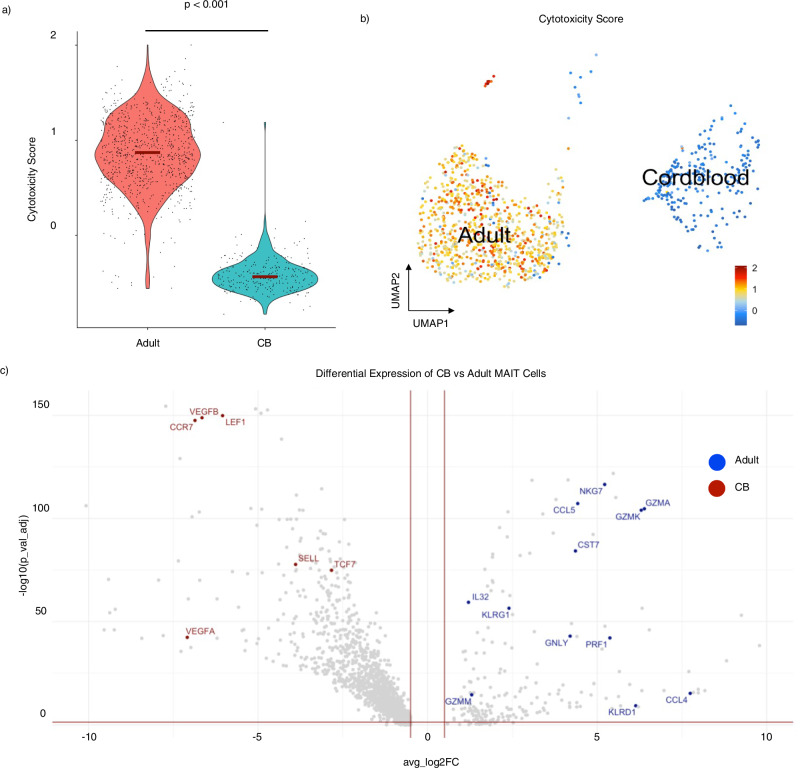
Adult MAIT cells are more cytotoxic than CB MAIT cells. **a**–**b** Cells were sorted for TRAV1-2( + ) MR1-restricted T (MR1T) cells and then cytotoxicity score (*PRF1, GNLY, NKG7, GZMA, GZMB, GZMH, GZMK* and *GZMM*) was assessed for adult compared to cord blood (CB) MR1T cells. *p*-value < 0.0001 calculated using Wilcoxon signed-rank test. **c** Volcano plot for differential gene expression of CB mucosal-associated invariant T (MAIT) vs adult MAIT cells. *p*-values were calculated using the Wilcoxon signed-rank test with Bonferroni adjustment for multiple comparisons. Labeled genes were greater than 0.5 or less than −0.5 log fold change and had a Bonferroni-adjusted *p*-value of <0.05.

Since there is an expansion of MAIT cells over the first year of life, one possible explanation for the increased cytotoxicity expression profile demonstrated by adult as compared to CB MR1T cells (Fig. 1g), is the increased proportions of MAIT cells in adults compared to neonates. To explore this, we compared the cytotoxicity scores of the TRAV1-2(+) cell subset from CB to that of adult participants (Fig. 5a, b). Adult MAIT cells had much higher cytotoxicity scores compared to CB MAIT cells suggesting that even the CB MAIT cell subset of MR1T cells demonstrate decreased cytotoxicity as compared to adult MAIT cells. This is also reflected in the differentially expressed genes between CB MAIT and adult MAIT cells (Fig. 5c, Supplemental Data 3), wherein adult MAIT cells upregulated cytotoxicity and effector genes (*GZMA, GZMB, GZMK, GZMM, GNLY, PRF1, NKG7, CST7, KLRD1, KLRG1, CCL4, CCL5, IL32*) and downregulated naïve and repair genes (*LEF1, SELL, CCR7, TCF7, VEGFA, VEGFB*). Furthermore, differential gene expression between adult and CB-derived TRAV1-2(-) MR1T cells, demonstrates that adult TRAV1-2(-) MR1T cells also had upregulation of many cytotoxic and effector genes (CCL4, CCL5, GZMA, GZMH, KLRBA, CST7 etc.), whereas CB TRAV1-2(-) MR1T cells had upregulation of many naïve associated genes (CCR7, SELL, TCF7; Supplemental Fig. 5). This suggests that CB MR1T cells continue to mature in the periphery after birth.

### Increased phenotypic diversity of CB MR1T cell clones as compared to adult MR1T cell clones

**Fig. 6:**
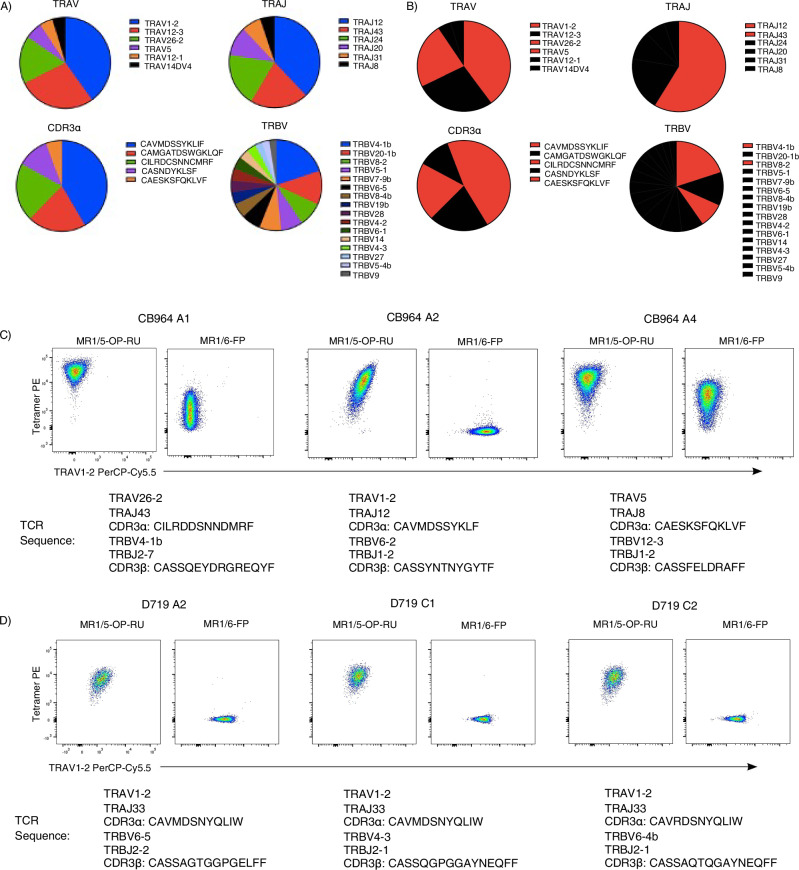
Three CB MR1T cell clones are largely representative of the diverse TCR repertoire of a neonate from whom a CB specimen was derived. **A** TCR repertoire of donor CB964 as determined by single cell TCR sequencing (scTCR-seq) from which cord blood (CB) MR1-restricted T (MR1T) cell clones were isolated. **B** Red coloring represents the TCRs expressed by the three CB MR1T cell clones that overlap with the TCR repertoire as determined by scTCR-seq of the MR1/5-OP-RU tetramer(+) cell population from CB964. **C** MR1/5-OP-RU and 6-FP tetramer staining and analysis by flow cytometry of CB MR1T cell clones and TCR sequence obtained by scTCR-seq for CB964 A1, CB964 A2 and CB964 A4. **D** MR1/5-OP-RU and 6-FP tetramer staining and analysis by flow cytometry of adult MR1T cell clones and TCR sequence obtained by scTCR-seq for D719 A2, D719 C1 and D719 C2.

MR1T cell clones were isolated by limited dilution assay (LDA) of MR1/5-OP-RU tetramer (+) cells derived from one adult participant (D719) and one CBMC specimen (CB964). Herein, we characterize two CB TRAV1-2(-) MR1T clones (CB964 A1/A4), one CB MAIT clone (CB964 A2) and three adult MAIT clones (D719 A2/C1/C2). We first validated that clones isolated from CB964 largely represent the diversity of TCR usage seen in this CB specimen. Figure 6a shows the TCR diversity of the ex vivo MR1/5-OP-RU tetramer(+) cell population from CB964 as determined by scTCR-seq and Fig. 6b shows the TCR usage of the three clones isolated from CB964, illustrating that these T cell clones isolated using LDA are largely representative of the diverse TCR repertoire of the participant from whom this CB specimen was derived. The tetramer staining and TCR usage of the MAIT and TRAV1-2(-) CB clones and the three adult MAIT clones, are shown in Fig. 6c and Fig. 6d, respectively. As expected, all MR1T cell clones retained strong MR1/5-OP-RU staining. MR1/6-formylpterin (MR1/6-FP) did not stain the MAIT cell clones, while both TRAV1-2(-) CB clones demonstrated MR1/6-FP staining. CB964 A1 uniformly expressed CD8 and variably expressed CD4 (Supplemental Fig. 5a). CB964 A2 and A4 expressed both CD8 and CD4 (Supplemental Fig. 6b, c). Each of the adult MAIT clones utilized TRAV1-2/TRAJ33 (Fig. 6d) and were CD8( + )CD4(-) (Supplemental Fig. 6d–f).

### CB MR1T cell clones demonstrate different unstimulated and stimulated gene expression profiles from adult MR1T cell clones

**Fig. 7:**
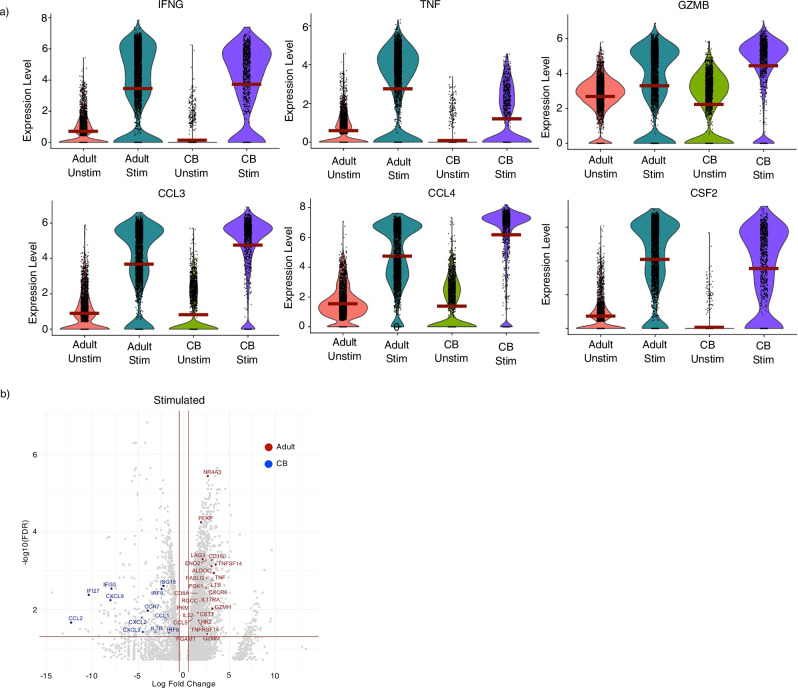
CB MR1T cell clones can produce typical inflammatory/cytotoxic cytokines by gene expression, but have distinct gene expression profiles from adult MR1T cell clones. **a** Violin plot of gene expression of common inflammatory/cytotoxic genes from stimulated (PMA/Ionomycin) and unstimulated MR1-restricted T (MR1T) cell clones from an adult compared to those isolated from cord blood (CB; CB964). IFNG = IFN-γ; GZMB = granzyme B. **b** Volcano plot of differentially expressed genes of adult compared to CB MR1T cell clones when stimulated with PMA/Ionomycin. *p*-values were calculated using the Wilcoxon signed-rank test with Bonferroni adjustment for multiple comparisons. Horizontal red line represents Bonferroni-adjusted *p*-value of 0.05, and vertical red lines represent log fold change of 0.5 or −0.5.

To test the functional response of CB MR1T cells compared to adults, PMA/ionomycin stimulated and unstimulated clones were analyzed by scGEX-seq. Similarly to adult MAIT cell clones, CB MAIT and TRAV1-2(-) MR1T cell clones produced pro-inflammatory and cytotoxic cytokines following stimulation (Fig. 7a, Supplemental Fig. 7). Differential gene expression comparing CB and adult clones showed many significantly differentially expressed genes both unstimulated (Supplemental Fig. 8) and following stimulation (Fig. 7b, Supplemental Data 4, 5). Notable trends include unstimulated CB MR1T cell clones have increased expression of naïve genes (*FUOM, SELL, CCR7, IL7R, LEF1, RGS10*), along with increased expression of many chemokines (*CCL3, CCL4, CCL23, CCL18, CCL1, CCl22, CCL24, CCL8, CCL13, CCL7, CCL2, CXCL9, CXCL12, CXCL3, CXCL16*) and increased type I interferon genes (*IFI30, IFI27, IFI44L, IFIT3, IFIT1, IFI6, IFI27L1, ISG15, MX2, IRF4, IRF9, IRF8, HERC3*) as compared to unstimulated adult MAIT cells clones (Supplemental Fig. 8). Unstimulated adult MAIT cell clones show increased expression of cytotoxic genes (*GZMH, PRF1, LTB, FASLG*) along with glycolysis genes (*PGAM5, ALDOC, PFKP, PGK1, ENO1, ENO2, GAPDH, PKM*), associated with effector phenotype in MAIT cells as compared to unstimulated CB MR1T cell clones^34^. Following stimulation, CB MR1T cells continued to have increased chemokines and type I interferon genes expression relative to adult MAIT cell clones, while adult MAIT clones demonstrated activation markers (*CD8A, TNFRSF14, NR4A3, FASLG, LTB, RGCC, CST7*), pro-inflammatory cytokines (*IL26, IL32, TNF*), glycolysis genes (*PFKP, ENO2, PGK1, PKM, HK2, ALDOC*) and exhausted T-cell genes (*LAG3, CD160, CTLA4, CD244*) as compared to CB MR1T cell clones (Fig. 7b).

### CB MR1T cell clones demonstrate differential recognition of childhood pathogens

We next tested CB MR1T cell clones against a panel of common childhood pathogens (*E. coli, S. aureus, S. pneumoniae)* as well as various mycobacteria (*M. tuberculosis* auxotroph; mc^2^6206 [H37Rv ΔpanCD ΔleuCD]^35^, *M. smegmatis*), and mycobacterial MR1T ligands (photolumazine 1 [PL1], deazalumazine [DZ])6^5^ and compared this to an adult MAIT clone; D426 G11^6^. All of these bacteria/mycobacteria are riboflavin producers and thus should activate MR1T cells.

**Fig. 8:**
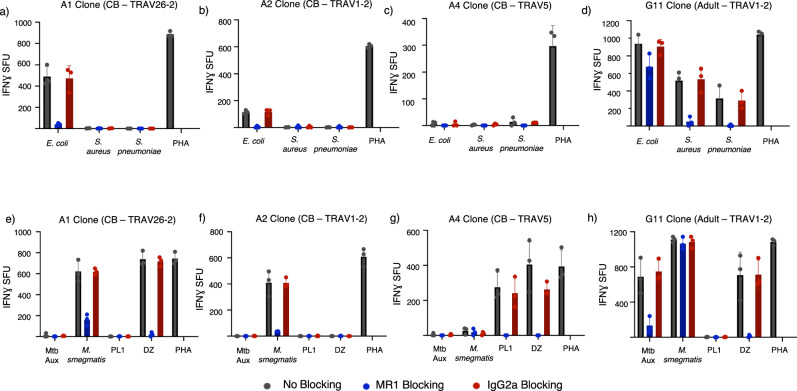
CB and adult MR1T cell clones have distinct and diverse microbial recognition patterns. **a**–**d** IFNγ ELISpot of cord blood (CB) MR1-restricted T (MR1T) cell clones (A1, A2 and A4) and adult MR1T cell clone (G11) responses to common childhood bacterial pathogens. Responses without blocking, anti-MR1 blocking antibody or isotype control blocking antibody are represented. Each clone was tested against dendritic cells (DCs) without microbes or antigens added, and this background was subtracted from IFNγ Spot Forming Units (SFU) shown. 3 biological replicates were done on different days, each with 2 technical replicates done per experiment. **e**–**h** IFNγ ELISpot of CB MR1T cell clones (A1, A2 and A4) and adult MR1T cell clone (G11) responses to mycobacteria and mycobacterial antigens. Responses without blocking, anti-MR1 blocking antibody or isotype control blocking antibody are represented. Three biological replicates were done on different days, each with 2 technical replicates done per experiment. SFU = spot forming units. PHA = phytohaemagglutin P. PL1 = photolumazine I. DZ = deazalumazine.

Each clone had a very different pattern of microbe and antigen recognition by IFNγ ELISpot (Fig. 8a–h) and this recognition was blocked by anti-MR1-antibody, demonstrating MR1-dependence. While adult-derived G11, demonstrated strong responses to *E. coli, S. aureus*, and *S. pneumoniae* (Fig. 8d), none of the CB MR1T clones recognized *S. aureus* or *S. pneumoniae* and recognized *E. coli* less well (CB964 A1/A2; Fig. 8a, b), or not at all (CB964 A4; Fig. 8c). Regarding mycobacteria, the adult-derived clone, G11, responded to Mtb auxotroph (Fig. 8h), while none of the CB MR1T cells recognized Mtb auxotroph (Fig. 8e–g). G11 demonstrated a robust response to *M. smegmatis* (Fig. 8h), such that MR1 blocking was not apparent until using lower concentrations of the microbe (Supplemental Fig. 9) and selectively recognized DZ but not PL1 as previously reported^5^. CB964 A1 similarly recognized *M. smegmatis* and DZ but not PL1 (Fig. 8e). By comparison CB964 A2 robustly responded to *M. smegmatis* in an MR1-dependent manner but did not recognize PL1 or DZ (Fig. 8f), while CB964 A4 did not recognize *M. smegmatis* despite strongly recognizing PL1 and DZ (Fig. 8g). Thus, these three CB MR1T cell clones each expressing different TRAV’s, demonstrate differential recognition of *M. smegmatis* and known mycobacterial MR1T ligands.

### Three CB MR1T TCRs displayed different affinities for MR1 compared to two adult MAIT TCRs

**Fig. 9:**
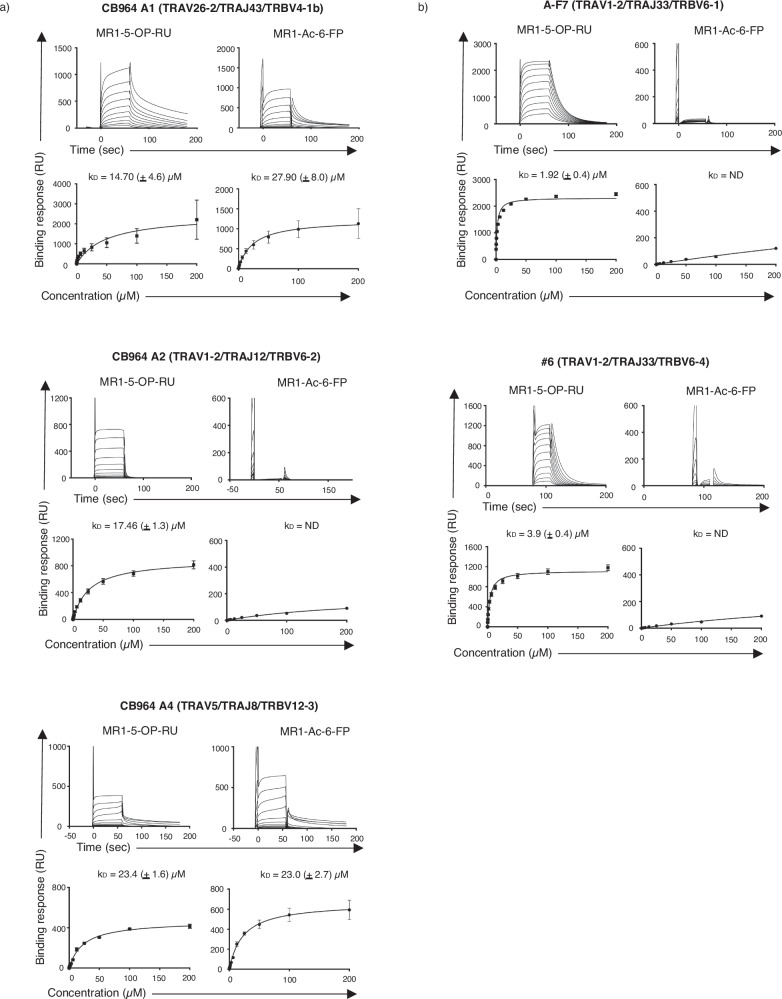
Steady-state affinity measurements of soluble TCRs for MR1-antigen complexes. The affinity of TCR-MR1-Ag interactions were determined using Surface Plasmon Resonance (SPR), by measuring the binding of various concentrations of soluble TCRs from **a** Cord blood (CB) MR1-restricted (MR1T) cell clones, CB926 (A1, *top*; A2, *middle*; A4, *bottom*) or from **b** adult mucosal-associated invariant T (MAIT) cell TCRs (A-F7, *top*; #6, *bottom*) against human MR1 refolded with 5-OP-RU and Ac-6-FP antigens. The SPR runs were conducted in duplicate in two independent experiments using different batches of proteins. Experiments were conducted with serial dilutions of the TCRs. The SPR sensograms (*upper panels*), equilibrium curves (*lower panels*) and steady state *K*_D_ values (µM) were prepared in GraphPad Prism 10. Error bars represent the mean and SD from technical replicates (two independent experiments). ND = not determined. RU = response unit.

To establish how adult and CB MAIT TCRs bind to MR1, three soluble extracellular domains of the adult and CB MR1T TCRs were expressed as inclusion bodies in *E. coli*, refolded into their native conformation and purified^36^. This included three CB TCR clones: CB946 A1 (TRAV26-2/TRBV4-1b), CB946 A2 (TRAV1-2/TRBV6-2) and CB946 A4 (TRAV5/TRBV12-3), as well as two adult MAIT TCRs: A-F7 (TRAV1-2-TRBV6-1), #6 (TRAV1-2-TRBV6-4)^37^. Next, Surface Plasmon Resonance (SPR) experiments were performed to examine the specificities and determine the steady state binding affinities (K_D_) of these TCRs against MR1-5-OP-RU or MR1-Ac-6-FP complexes (Fig. 9). As previously reported, the adult MAIT A-F7 and #6 TCRs bind only to MR1-5-OP-RU with high affinity (K_D_ ~ 2-4 µM; Fig. 9b)^36^, while the CB (A1, A2 and A4) TCRs exhibited a roughly 5-fold lower affinity of K_D_ ~ 15-24 µM to MR1-5-OP-RU (Fig. 9a). Notably, CB MR1T A1 and A4 bound similarly to MR1-Ac-6-FP (K_D_ ~ 23 µM), while CB MAIT A2 TCR clone did not bind to MR1-Ac-6-FP (Fig. 9a), correlating with the MR1/6-FP tetramer binding by flow cytometry (Fig. 6c). As such, the CB MR1T TCRs tested were MR1 reactive yet exhibited lower affinity towards MR1-5-OP-RU.

### Overview of CB MAIT TCR-MR1-5-OP-RU ternary complex

**Fig. 10:**
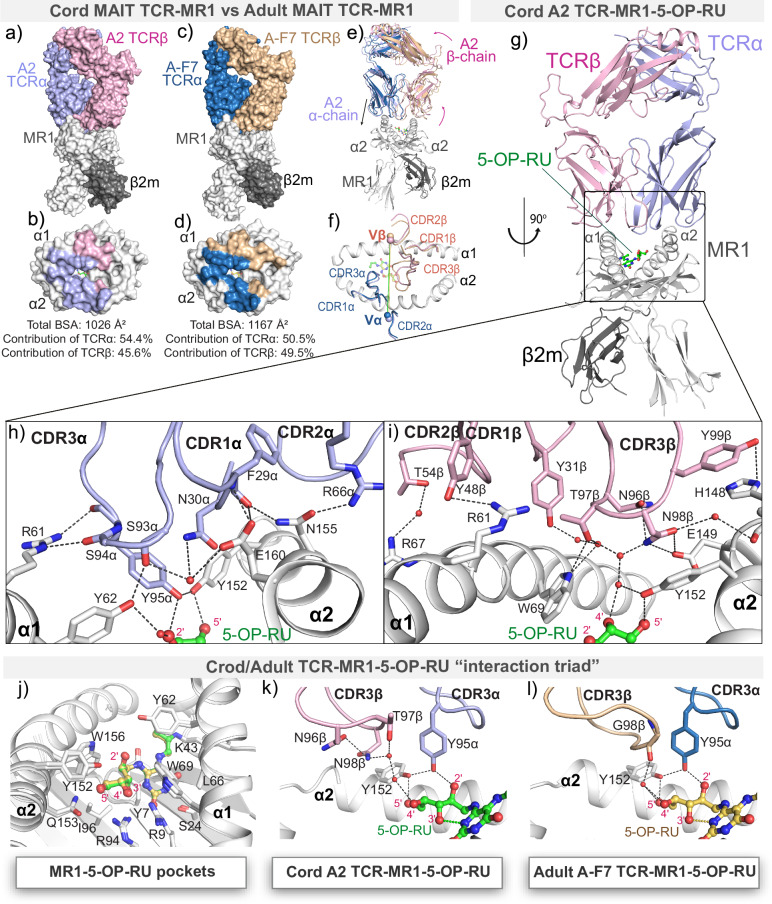
Structural comparison of ternary complexes of cord blood A2 and adult AF-7 typical MAIT TCRs with MR1-5-OP-RU. **a**–**d** Crystal structures of ternary complexes of (**a**–**b**) cord blood A2 mucosal-associated invariant T (MAIT; TRAV1-2/TRBV6-2) TCR-MR1-5-OP-RU and (**c**–**d**) adult MAIT A-F7 (TRAV1-2/TRBV6-1) TCR-MR1-5-OP-RU (PDB ID: 6PUC). The top panels (*a and c)* are molecular surface representations of the ternary complexes; the lower panels (**b** and **d**) illustrate the respective TCR footprints on the molecular surface of MR1-5-OP-RU. The MR1 and β2-microglobulin molecules are colored white and dark gray, respectively and 5-OP-RU is presented as green and yellow sticks in A2 and A-F7 ternary structures, respectively. A2 TCRα, lavender; A2 TCRβ, light-pink; A-F7 TCRα, sky-blue; A-F7 TCRβ, wheat. The atomic footprint is colored according to the TCR chain making contact. **e** Comparison of cord A2 TCR ternary complex relative to the adult A-F7 TCR docking positions. Arrows illustrate TCR rotation around the center of mass of the MR1, as well as displacement of the β-chain along the MR1 binding cleft. **f** Superposition of the CDR loops of cord MAIT A2 and adult MAIT A-F7 TCRs sitting atop MR1. The center of mass of the respective TRAV and TRBV variable domains are shown as spheres in the same colors as the respective variable domains. **g** Cartoon representation of the ternary structure of A2 TCR-MR1-5-OP-RU. Interactions between (**h**) the CDRα loops, **i** CDRβ loops of the cord A2 and MR1 residues. The interacting residues are represented as sticks. Hydrogen bond interactions are represented by black dashes. See also Supplemental Table 2. **j** Comparison of the MR1 antigen-binding pocket and the position of MR1 residues in both the cord A2 and adult A-F7 ternary structures. Interactions of the CDR3α and CDR3β of the cord A2 (**k**) and adult (**l**) MAIT TCRs, depicting the ‘interaction triad’ between Tyr152 of MR1, Tyr95α of CDR3α and 5-OP-RU.

We next determined the crystal structure of the CB964 A2 (TRAV1-2/TRBV6-2) TCR-MR1-5-OP-RU ternary complex at 2.5 Å resolution and compared it with the published adult MAIT A-F7 (TRAV1-2-TRBV6-1) TCR-MR1-5-OP-RU structure^36,38^ (Fig. 10a–d, Supplemental Table 1). The 5-OP-RU ligand in the CB964 A2 TCR-MR1-5-OP-RU complex was sequestered within the A′ pocket and formed a Schiff-base covalent bond with MR1-Lys43, consistent with all published adult MAIT TCR-MR1-5-OP-RU complexes^39^. The CB964 A2 TCR docked orthogonally atop the MR1 antigen binding cleft, with the TCR α- and β-chains positioned atop the α2- and α1-helices of MR1, respectively (Fig. 10a,b). The buried surface area (BSA) at the interface between CB964 A2 TCR and MR1-5-OP-RU was approximately ~1030Å^2^, a value that falls close to the range of BSA of adult MAIT TCR-MR1-5-OP-RU complexes (1060-1200Å^2^). Here, the center of gravity of the CB964 A2 α and β-chains were similar to the corresponding chains of the A-F7 TCR (Fig. 10e, f). While the α- and β-chains of adult MAIT TCRs contributed almost equally to the BSA of the interfaces of most TCR–MR1-Ag complexes, the α- and β-chains of the CB964 A2 TCR contributed to ~54.4% and 45.6%, respectively (Fig. 10b, d), with this difference being attributed to slightly differing MR1 docking modality of the CB964 A2 TCR β-chain (Fig. 10e, f). Specifically, the CB964 A2 TCR was slightly rotated in comparison to adult MAIT TCRs, which positioned the β-chain slightly away from MR1 and reduced the contribution of CB964 A2 TCR β-chain to the interface with MR1. Consequently, the CB964 A2 TCR adopted a moderately different footprints on MR1 compared to adult TCRs (Fig. 10b, d).

### Conserved recognition of riboflavin-derived 5-OP-RU by a CB and adult MAIT TCRs

The TRAV1-2 TCR α-chain docks similarly in both CB964 A2 and adult A-F7 TCRs, contributing to ~560Å^2^ and 590Å^2^ to the BSA of the interface of CB964 A2 TCR-MR1 and adult TCR-MR1 complexes, respectively. The TRAV1-2-TRAJ12 α-chain of the CB964 A2 TCR comprises a conserved set of TRAV1-2-encoded CDR1α residues; Gly28α, Phe29α and Asn30α and CDR2α residues; Val50α and Leu51α. These residues mediate conserved polar and van der Waals (vdw) interactions with residues of MR1 α2-helix, such as His148, Asn155 and Glu160 (Fig. 10g, h, Supplemental Table 2). Additionally, the TRAJ12-encoded CDR3α motif residues, ^93^Ser-Ser-Tyr-Lys^96^, of the CB964 A2 TCR forms conserved polar interactions with MR1-His58, Arg61, Tyr62, Tyr152, and Trp156 (Fig. 10h). Akin to the adult A-F7 TCR-MR1-5-OP-RU complex, the conserved Tyr95α of the CB964 A2 TCR formed a hydrogen bond with 2′OH group of 5-OP-RU and Tyr152 of MR1 α1-helix, thus forming the *‘interaction triad’* (Fig. 10j–l). The positioning of the ribityl tail of 5-OP-RU was conserved in both structures and governed by polar interactions mediated by MR1 residues, Arg9, Arg94, Tyr152 and Gln153 (Fig. 10j). Collectively, the CB964 A2 TCR α-chain TRAV1-2 interactions with MR1 were conserved when compared to the published adult MAIT TCR footprints on MR1^14,37^.

The TRBV6-2 β-chain of the CB964 A2 TCR contributed to ~470Å^2^ of the BSA of TCR-MR1 complex, but the TRBV6-1 β-chain of the adult TCR-MR1 complex was more closely associated with MR1 (BSA ~ 580Å^2^). Indeed, the TRBV6-1 of the adult A-F7 TCR is oriented closer to the MR1 α1-helix than the CB964 A2 TCR. As a result, it produces more CDRβ-MR1 mediated contacts, which affects their BSA to MR1 (Fig. 10b, d, f). Here the CB964 A2 CDR3β loop was essentially the main β-chain participant to the complex interface, followed by CDR2β, whereas the CDR1β loop was not involved in polar interactions with MR1 (Fig. 10i). Specifically, the Asn98β and Tyr99β of the CB964 A2 CDR3β loop formed hydrogen bonds with Glu149 and His148 residues of MR1 α2-helix respectively, whereas the Tyr48β of the CDR2β motif formed hydrogen bond with Arg61 and vdw contacts with Arg61 and Gln64 of the MR1 α1-helix (Fig. 10i, Supplemental Table 2). In accordance with these structural results and SPR affinity data, the CB964 A2 MAIT TCR can recognize riboflavin-derivative-based 5-OP-RU presented by MR1, despite having a weaker interaction with MR1 than adult MAIT TCRs.

## Discussion

Understanding MR1T cell development and early life capacity could help elucidate mechanisms of enhanced susceptibility to infection in neonates and inform strategies to mitigate this risk. Here, we compare ex vivo MR1/5-OP-RU tetramer(+) cells from CB and adult participants, demonstrating that ex vivo CB-derived MR1T cells exhibit a lower cytotoxicity and inflammatory gene expression profile, accompanied by a much more diverse TCR repertoire. Stimulation of CBMC with 5-OP-RU in vitro led to selective expansion of MAIT cells over TRAV1-2(-) MR1T cells, suggesting 5-OP-RU may drive MAIT cell expansion in early life. Notably, CB-derived MAIT and TRAV1-2(-) MR1T cell clones failed to respond to several common childhood pathogens (*S. aureus, S. pneumoniae, M. tuberculosis*), despite their riboflavin production. Crystal structure analysis of a CB MAIT TCR-MR1-5-OP-RU complex compared to an adult-derived MAIT TCR revealed that the β-chain binding reduced TCR affinity for an MR1 ribityl ligand. This represents a comprehensive transcriptional profile of CB-derived MR1/5-OP-RU tetramer(+) MR1T cells, including TRAV1-2(-) subsets, and a detailed analysis of the capacity of these cells to recognize microbes and microbial MR1 ligands. These findings enhance our understanding of MR1T cell development in early life and their role in neonatal immune susceptibility.

It has previously been shown that MAIT cells, defined as TRAV1-2 + , CD161 high cells, acquire anti-microbial activity, including the ability to produce IFNγ in utero, prior to the development of the microbiota and thus prior to microbial antigen exposure^40^. We have previously shown that at birth, the majority of MR1T cells are in fact not MAIT cells and are instead TRAV1-2(-) MR1T cells and that functional MR1T cells as defined by TNF production to *M. smegmatis*, were exclusively contained in the MAIT cell subset^20^. We have also recently explored this development in more detail, looking at the specific development of MAIT cells compared to TRAV1-2(-) MR1T cells in CB, 10-week-old and adult participants^41^. In that work, we found that by 10-weeks MAIT cells had upregulated cytotoxic genes and downregulated naïve genes, whereas this shift was not seen until adulthood for TRAV1-2(-) MR1T cells, suggesting that maturation of TRAV1-2(-) cells happens after 10 weeks of age. In addition, MAIT cells rapidly expand in early life, whereas TRAV1-2(-) MR1T cells persist as a relatively fixed proportion of all T cells in adult populations. Taken together, these data support the idea that MR1T cells, as comprehensively defined by positive MR1/5-OP-RU tetramer staining in CB, have diminished capacity to recognize microbes because MAIT cells are relatively infrequent. Our work similarly finds that in CB, MAIT cells upregulate genes that suggest that these cells could be more functional compared to TRAV1-2(-) MR1T cells. We extend this work, however, finding that even among CB MAIT cells there is higher expression of naïve genes, and lower expression of cytotoxicity genes compared to adult MAIT cells. This includes upregulation of *GNLY*, which has recently been suggested to mark MAIT cells that are cytotoxic^42^. These findings are similar to recently published work from Loh et al. comparing MAIT cells from thymic samples to adult PBMC MAIT cells^43^. They similarly found that thymic MAIT cells had upregulation of naïve T cell genes compared to adult MAIT cells. By contrast, the cytotoxicity score was upregulated in adult MAIT cells as compared to thymic MAIT cells.

Therefore, at birth MAIT cells are not only less frequent, but also less mature, demonstrating less potential for functional capacity than adult MAIT cells. This maturation over life would therefore most likely happen in the periphery in response to environmental exposures after birth. Conversely, we believe that the TRAV1-2(-) MR1T cells persist in adults at frequencies comparable to other T cell subsets. Furthermore, we speculate that the TRAV1-2(-) MR1T cells that persist into adulthood, are not unlike naïve conventional T cells that persist into adulthood, in that these cells have not yet experienced activating ligands. For example, we previously demonstrated that a TRAV-12( + ) MR1T cell clone isolated from an adult recognizes *S. pyogenes*, which doesn’t produce 5-OP-RU, and poorly recognizes riboflavin producing organisms^6^. This is further supported by work from Harriff et al. demonstrating an unexpectedly diverse MR1 ligandome^5^.

Furthermore, our results confirm and extend the prior characterization of human MR1T cell development. CB-derived MR1T cells had significantly higher expression of genes associated with naïve cells and stage 2 MR1T cells than did adult MR1T cells. This is in keeping with previously published work that explored MR1/5-OP-RU tetramer(+) TRAV 1-2(+) cells, from CB, showing that the majority are stage 2 or early stage 3^44^. *LEF1* in particular has been shown to be a marker of early stage 3 MAIT cells^45^, and is also found to be significantly upregulated in CB-derived TRAV1-2(-) MR1T cells and also CB MAIT cells, as compared to adult MR1T cells. This further supports that immature TRAV1-2(-) MR1T cells and MAIT cells are present in the CB of humans and that extrathymic maturation of these cells occurs after birth. The selective expansion of MAIT cells we found following 5-OP-RU stimulation, suggests that exposure to 5-OP-RU following birth could represent a driving force for this shift from predominantly TRAV1-2(-) MR1T cells to predominantly MAIT cells later in life. This is supported in murine models showing that establishment of mucosal colonization of MAIT cells is dependent on intestinal colonization of 5-OP-RU producing microorganisms^21,46^.

We have previously demonstrated that at birth, there is significantly more α-chain diversity in the TCR repertoire of MR1T cells^20^. Herein, we confirm and extend this finding, observing significantly more diversity within the entire TCR repertoire of MR1T cells at birth. Among CB MAIT cells there is increased α-chain diversity as evidenced by decreased canonical TRAV1-2/TRAJ33, TRAV1-2/TRBV6-4 and TRAV1-2/TRBV20-1 pairings. Among TRAV1-2(-) MR1T cells, we observe no skewing of either α- or β-chain usage. This degree of TCR diversity among the TRAV1-2(-) MR1T cells is similar to that observed among conventional MHC1a-restricted T cells^47^. We speculate that TRAV1-2(-) MR1T cells may have the capacity to respond to cognate antigenic exposure, albeit the full repertoire of ligands recognized remains to be determined. Therefore, while we find tremendous TCR diversity among MR1T cells at birth, the functional consequences of this remains largely unexplored. Among MAIT cells, the TCR β-chain can contribute to MR1 ligand discrimination^38,48–50^. Crystal TCR structure from the CB MAIT clone, showed that although it bound 5-OP-RU, it did so with a much lower affinity than would be expected for most MAIT cells, with the α-chain binding 5-OP-RU in a conventional manner, but with less β-chain interaction than is typical. This adds to the previous literature suggesting a role of the β-chain in fine-tuning the MAIT cell antigen response and provides a structural basis for why the β-chain interaction with MR1 is important for TCR recognition of microbial ligands^37,48,49^.

To study the pathogen recognition pattern and functional capacity of CB MR1T cells, we studied three MR1T cell clones isolated from CB of one individual. While we acknowledge that these T cell clones likely do not fully represent either this individual’s MR1T cell population nor neonatal MR1T cell populations in general, these T cell clones demonstrated similar diversity of CD4/CD8 and TCR expression to this neonate’s ex vivo MR1T cell population as examined by scTCR-seq. Moreover, the gene expression profile of the three unstimulated cord blood clones was similar to that observed in the ex vivo MR1T cell data derived from five CB specimens. Cloning MR1T cells from CB allows detailed examination of TCR recognition of MR1 ligands. Despite that all CB-derived clones stained positive for the MR1/5-OP-RU tetramer, none of these clones responded to some common riboflavin-producing childhood pathogens, *S. aureus*, *S. pneumoniae* and *M. tuberculosis*. The reasons for this discrepancy are not entirely clear, but there are several possible explanations. As among MAIT cells, the TCR β-chain can contribute to MR1 ligand discrimination^37,48,49^, one possibility is that the less typical β-chain contacts for the CB MAIT cell clone TCR contributes to the lack of reactivity towards these riboflavin-producing pathogens. Another possibility is that the concentration of ligand processed and presented during the infection with these pathogens could be insufficient to activate these clones. Supporting this, we see that one of the CB T cell clones responds to PL1, which can be found in *M. smegmatis* supernatants^5^. However, we do not know the extent to which it is produced by live *M. smegmatis* and hence the quantity of this ligand may well be insufficient to activate our T cell clone. In addition, we have recently shown that host cell riboflavin and its catabolites can bind to MR1, reducing MR1 expression at the cell surface and inhibiting MAIT cell activation by pathogen-derived MR1 ligands^5,50^. Another possibility is that the optimal ligands for the TCRs expressed by CB-derived MR1T cell clones are not produced by these organisms. In this regard, for adults, 5-amino-6-D-ribityaminouracil (5-A-RU) is a key precursor for the production of MAIT cell ligands^51^, but this may not be the case for the diverse TCRs expressed by CB MR1T cells. It is therefore possible that these CB TCRs could recognize 5-OP-RU, but not a sufficiently activating ligand produced by these particular riboflavin-producing organisms. While the generalizability of these results from three CB MR1T cell clones from one individual to neonatal MR1T cells in general is limited, we speculate that the diminished response of neonatal MR1T cells to common childhood pathogens, could reflect a hole in the repertoire of the MR1T cells’ response to these organisms at birth and that this defect is repaired by environmental exposures after birth.

It has previously been suggested that TRAV1-2(-) MR1T cells recognize self-antigens, in the absence of microbes^18^. Here, we find that neither of the two TRAV1-2(-) MR1T clones tested showed evidence of response to self. Furthermore, we find that one of the TRAV1-2(-) MR1T clones was able to recognize *E. coli*. Therefore, as shown previously^6^, we find that TRAV1-2(-) MR1T cells can also recognize microbes. TRAV1-2(-) MR1T cells persist into adulthood, and as autoreactive TRAV1-2(-) MR1T cells must necessarily survive thymic selection, we postulate that the expansion of these cells must occur later in life, as might also occur with microbially reactive TRAV1-2(-) MR1T cells.

MAIT cells play an important role in defense against many microbial infections, including many common bacterial causes of childhood infections^4,16^. Our results are consistent with that the decreased frequency of functional MR1T cells at birth could play a role in susceptibility to bacterial infection in early life. However, we do also find both MAIT and TRAV1-2(-) MR1T cells at birth that are microbial responsive and have effector abilities when stimulated and therefore could be especially critical in early life prior to the development of classical adaptive immune responses^52^. The shift in TCR repertoire from CB to adults, suggests that exposures after birth shape these cells to become more functional, and therefore suggests that these cells could be harnessed in early life for the development of novel childhood vaccines.

## Methods

### Study participants

This study was conducted according to the principles expressed in the Declaration of Helsinki. Study participants, protocols, and consent forms were approved by the Institutional Review Board at Oregon Health & Science University (OHSU; IRB00000186 and IRB#00005032). All ethical regulations relevant to human research participants were followed. For adult participants, they were recruited via IRB-approved posted flyers and written informed consent was obtained. Adult participants were mixed male (*n* = 2) and female (*n* = 3), based upon self-reporting. Obtaining informed consent for deidentified CB samples was waived by the Institutional Review Board at Oregon Health & Science University. Sex was not reported for the neonates from whom cord blood samples were obtained as these samples were de-identified. Umbilical CB was obtained from the placenta of uncomplicated term pregnancies at OHSU in Portland, Oregon, after delivery into citrate CPT tubes (BD, 362761). Blood was processed per manufacturer’s instructions within 24 h of collection, and the resulting CB mononuclear cells (CBMC) were cryopreserved. Adult peripheral blood mononuclear cells (PBMC) were obtained via leukapheresis from healthy adult participants and cryopreserved. Adult participants lacked any detectable responses to Mtb immunodominant antigens, CFP-10 and ESAT-6 and were therefore assumed to be Mtb uninfected.

### Single-cell sequencing of MR1T cells and MR1T cell clones

The following reagents were obtained through the NIH Tetramer Core Facility: MR1/5-OP-RU tetramer and MR1/6-FP tetramer^38^. CB and adult MR1T cells were identified by positive staining with the MR1/5-OP-RU tetramer^11,20^. CBMC or PBMC were thawed in the presence of DNase, resuspended in 10% heat inactivated human serum (HuS) with RPMI (Gibco, 22400105) at a concentration of 2×10^7^/ml. CBMC/PBMC (2 ×10^6^ cells/well) were then stained with MR1/5-OP-RU tetramer at a dilution of 1:250 for 45 min at room temperature^20^. Cells were then subsequently stained with an antibody cocktail of surface stains (listed in Supplemental Table 3) for 30 min at 4 °C to allow non-CD3+ T cells or dead cells to be excluded. Cells were incubated in Human TruStain FcX (1:200 dilution, Biolegend, 422302) for 10 min at 4 °C and then stained with TotalSeq-C hashtag antibodies and CITE-seq antibodies (Biolegend) listed in Supplemental Table 4 for 30 min at 4 °C. Samples were then then sorted using a BD FACSAria III Cell Sorter for MR1/5-OP-RU tetramer positive cells (representative gating strategy shown in Supplemental Fig. 10) and all MR1/5-OP-RU tetramer-positive cells were considered MR1T cells. Sorted MR1T cells were then loaded into 10X Genomics Chromium Next GEM Single Cell 5’ v2. Full protocol details of single-cell GEX, TCR and cell surface protein library preparation can be found from the 10X genomics website (https://cdn.10xgenomics.com/image/upload/v1722286086/support-documents/CG000330_Chromium_Next_GEM_Single_Cell_5_v2_Cell_Surface_Protein_UserGuide_RevG.pdf). Finished libraries were then sent to Novogene Corporation Inc. in Sacramento California for NovaSeq 6000 sequencing. MR1T cell clones were also sequenced using 10X Chromium Next GEM Single Cell 5’ v2. Clones were either unstimulated at time of sequencing or were stimulated with PMA/Ionomycin (Millipore Sigma, P8139-5MG, I3909-1ML) for 3 h prior to sequencing.

### Single-cell RNA-seq pre-processing

Raw sequence reads were processed using 10X Genomics Cell Ranger software (version 6.1.1). The resulting sequence data were aligned to the GRCh38 human genome. Cell demultiplexing used a combination of algorithms, including GMM-demux, demuxEM and BFF, implemented using the cellhashR package^53–55^. Droplets identified as doublets (i.e., the collision of distinct sample barcodes) were removed from downstream analyses. We additionally performed doublet detection using DoubletFinder and removed doublets from downstream analysis^56^. Next, droplets were filtered based on UMI count (allowing 0–20,000/cell), and unique features (allowing 200–5000/cell). Additionally, we computed a per-cell saturation statistic for both RNA and antibody derived tags (ADT) data, defined as: 1 – (#UMIs / #Counts). This statistic provides a per-cell measurement of the completeness with which unique molecules are sampled per cell and has the benefit of being adaptable across diverse cell types. Data were filtered to require RNA saturation > 0.35. Analyses utilized the Seurat R package, version 4.2^57^. Using standardized methods implemented in the Seurat R package, counts and UMIs were normalized across cells, scaled per 10,000 bases, and converted to log scale using the ‘NormalizeData’ function. These values were then converted to z-scores using the ‘ScaleData’ command. Highly variable genes were selected using the ‘FindVariableGenes’ function with a dispersion cutoff of 0.5. Principal components were calculated for these selected genes and projected onto all other genes using the ‘RunPCA’ and ‘ProjectPCA’ commands. Clusters of similar cells were identified using the Louvain method for community detection, and UMAP projections were calculated. CITE-seq data were CLR-normalized by lane, meaning raw count data are subset per lane, CLR normalization performed (as implemented in the Seurat R package, using margin = 1), using all QC-passing cells/lane. Normalization per-lane was performed to reduce batch effects.

### TCR sequence analysis

Raw sequence reads for gene expression and TCR enrichment were first processed using cellranger software, version 6.1.1 (10X Genomics). The raw clonotype calls produced by cellranger vdj were extracted from the comma-delimited outputs. Cells were demultiplexed and TCR calls were assigned to samples using custom software, made publicly available through the cellhashR package, with the GMM-Demux, demuxEM, and BFF algorithms^53–55^. The clonotype data generated by cellranger were filtered to drop any cells where the TCR calls were lacking a CDR3 sequence, the clonotype was not marked as full-length, or the clonotype lacked a called V, J, or constant gene. Rows with chimeric V/J/C combinations (i.e., TRBV / TRAC) were filtered, with the exception that segments consisting of a TRDV/TRAC or TRAV/TRDC were permitted. These chimeric segments were classified according to the constant region chain. Data was analyzed in R studio using Seurat package^58^.

### Limited Dilution Cloning (LDC)

CBMC were stained with Propidium Iodide (Milteny Biotec, 130-093-233), MR1/5-OP-RU tetramer (NIH tetramer core), and an antibody cocktail of surface stains to sort out non-CD3+ cells (listed in Supplemental Table 3)^59^. Live, MR1/5-OP-RU tetramer positive cells were sorted and then cryopreserved. After thawing, the cells were plated in a 96-well plate in a limited dilution assay with 1.5×10^5^ irradiated PBMC (3000 cGray) and 3×10^4^ irradiated LCL (6000 cGray) along with αCD3 (30 ng/ml, eBioscience, 16003738) and IL-2 (2 ng/ml, Proleukin, NDC 73776-0022-01) in RPMI 1640 supplemented with 10% heat inactivated human serum in 96 well round bottom plates. On day 5, the αCD3 was washed out by removal of half of the volume from the wells and replaced with additional IL-2 supplemented media. Media was changed for all wells every 2-3 days and replaced with IL-2 supplemented media. T cell “buttons” were evaluated for growth on Day 20 and selected buttons stained with the MR1/5-OP-RU tetramer, CD3 PeCy7 (clone SK7; Biolegend, 344816), CD4 FITC (clone SK3; Biolegend, 344604), CD8 APC Cy7 (clone SK1; Biolegend, 344714), and TRAV1-2 BV605 (clone 3C10; Biolegend, 351720). T cell clones that remained MR1/5-OP-RU tetramer positive were re-expanded in T12.5 flasks with irradiated PBMC and LCL as well as RPMI 1640 media with 10% Human serum supplemented with αCD3 and IL-2 as above^3^.

### Clone phenotypic confirmation

Following LDC, isolated clones were analyzed phenotypically by flow cytometry to confirm retention of positive staining for the MR1/5-OP-RU tetramer and clonality. Clones were thawed in the presence of DNase (Roche, 10104159001), resuspended in 10% heat inactivated human serum with RPMI (Gibco, 22400105) and counted. Viable cells (10^5^ cells/well) from each clone were plated in duplicate into a 96 well plate. Clones were washed in 2% fetal bovine serum (FBS, Gemini, 100–106) in phosphate buffered saline (PBS, Gibco, 10010-072), followed by staining with either MR1/5-OP-RU tetramer or MR1/6-FP tetramer at a dilution of 1:500 for 45 min. Following this, cells were stained with surface stains (Supplemental Table 5) for 30 min. Cells were washed in PBS and then resuspended in 1% paraformaldehyde (Electron Microscopy Sciences, 1574100) and analyzed on a FACSymphony. Data was subsequently analyzed using FlowJo.

### Microbes and antigens

*E. coli, S. aureus, S. pneumoniae* were purchased from American Type Culture Collection (ATCC, 11775, 6538 P, 33400 respectively) and instructions for resuspension and growth were followed. Following growth of each bacteria, the concentration was calculated through serial dilutions and plating. Appropriate multiplicity of infection was determined through titrations using an adult-derived MAIT cell clone (D426 G11) to confirm adequate response to each bacterium. *M. tuberculosis* (Mtb) auxotroph mc^2^6206 (H37Rv ΔpanCD ΔleuCD)^35^ was as a kind gift from Bill Jacobs (MTA-IN19-168) and is modified such that it is only able to grow in the presence of externally supplemented pantothenate and leucine and thus can be handled in a biosafety level 2 setting. *M. smegmatis* is used frequently in our lab and two *M. smegmatis* MR1 ligands, photolumazine I (PL1) and deazalumazine (DZ), were previously generated and details of this have been previously published^5^.

### 5-OP-RU stimulation of CBMC

CBMC from three cord blood donors were thawed at Day 0 and a sample of 2e6 cells taken from each donor for initial flow phenotyping (Supplemental Table 5). The remaining cells were rested overnight in RPMI 1640 medium containing 10% heat-inactivated human serum. On Day 1 and 7, half of the CBMC were stimulated with 5-OP-RU (1 µM, OHSU Medicinal Chemistry Core) while the other half remained unstimulated. Starting at Day 1, all cells received recombinant IL-2 (5 ng/mL) with additional IL-2 added every 2–3 days. Cells were harvested and analyzed by flow cytometry on Day 14 (Supplemental Table 5).

### Microbe and antigen responses of MR1T cell clones

Microbe and antigen responses from MR1T cell clones were determined using an enzyme-linked immunosorbent spot (ELISpot) assay for IFN-γ^6^. Human DC were isolated from PBMC by plate adherence for 1 h, irradiated (3000 cGy) and cultured in RPMI + 10% HuS with 30 ng/ml GM-CSF (Leukine, NDC 0024-5844-01) and 10 ng/ml IL-4 (R&D Systems, BT-004-500) for 5 days^6^. ELISpot plates (Millipore Sigma, MSHAS4510) were coated with anti-IFN-γ antibody (Mabtech, clone 1-D1K, Cat No. 3420-3-1000, 10 μg/ml). After overnight incubation at 4 °C, ELISpot plates were washed three times with PBS and then blocked with RPMI 1640 + 10% human serum for at least 1 h. For experiments with Mtb, 5×10^5^ DC were plated in each well of an ultra-low adherence 24 well tissue culture plate. Mtb was grown fresh in 7H9 Middlebrook broth (BD, 271310), supplemented with 50 μg/mL of leucine and 24 μg/mL of pantothenate, grown to an optical density of 0.5, 40ul (MOI 9), added and incubated overnight. DC (1×10^4^ cells) were plated in the ELISpot wells in RPMI + 10% HuS. For experiments with *E. coli, S. aureus, S. pneumoniae* and *M. smegmatis*, PL1, and DZ, DC (1 × 10^4^ cells) were plated in the ELISpot wells in RPMI + 10% HuS. The bacteria or antigens were added to the wells at concentrations or MOI indicated for each experiment and incubated for 1 h. For all experiments, T cell clones (5000 cells) were added, and the plate was incubated overnight at 37 °C. IFN-γ ELISpots were enumerated following development with an alkaline phosphatase-conjugated secondary antibody (Mabtech, 7-B6-1-ALP, Cat No. 3420-9A-1000, 1:1000). ELISpot plates were washed extensively in PBS + 0.05% Tween-20 (Millipore Sigma, P2287), incubated with secondary antibody for at least 2 h at room temperature, washed in PBS + 0.05% Tween-20 again and then PBS before incubation with BCIP/NBT-plus developer (Mabtech, 365010). Dried plates were imaged with an AID ELISPOT reader and spots were counted using the AID ELISPOT software Version 8.0^59^. Antibody blocking was performed using the anti-MR1 26.5 clone (OHSU antibody core) and an IgG2a isotype control (Biolegend, MOPC-173, Cat No. 400224) added at 5 μg/ml for 1 h prior to the addition of ligand. All samples were done as technical replicates in duplicate, and three independent biological replicates were done on separate days. Negative and positive controls were also performed using T cells and DC without antigen or with PHA (10 μg/ml; Millipore Sigma, L-4144). Background spots seen in negative control wells were subtracted from the remainder of the plate. All ELISpot analysis and graphing were done in GraphPad Prism.

### Expression, refold and purification of MR1-β2 m and MAIT TCR proteins

Human A-F7 (TRAV1-2/TRBV6-1)^37^, #6 (TRAV1-2-TRBV6-4)^37^, and CB964 A2 TCR (TRAV1-2-TRBV6-2) MAIT TCR proteins were refolded from inclusion bodies at 4 °C for an overnight period using the refolding buffer that contained 0.1 M Tris pH 8.5, 6 M urea, 2 mM EDTA, 0.4 M L-arginine, 0.5 mM oxidized glutathione, and 5 mM reduced glutathione^37^. The wild-type or C-terminal cysteine mutated MR1-β2m was refolded at a 10x molar ratio of the investigated compound in the same refold buffer^38^. The refolded MR1-ligand and TCR proteins were purified using three sequential purification procedures: crude DEAE anion exchange, S200 15/60 size exclusion chromatography, and HiTrap-Q HP or MonoQ 10/100 GL anion exchange. The soluble CB946 A1 (TRAV26-2/TRBV4-1b) and CB946 A4 (TRAV5/TRBV12-3) TCRs were expressed in Expi293F GnTI- cells and the dialyzed proteins were purified by immobilized metal affinity chromatography, followed by Superdex 75 size exclusion chromatography. The protein purity was assessed using sodium dodecyl sulfate–polyacrylamide gel electrophoresis (SDS-PAGE) and quantified using a NanoDrop spectrophotometer.

### Surface Plasmon Resonance measurements

All Surface Plasmon Resonance (SPR) experiments were conducted at 25 °C, in duplicate (*n* = 3), on a BIAcore 3000 instrument using HBS buffer (10 mM HEPES-HCl pH 7.4, 150 mM NaCl, and 0.005% surfactant P20)^60^. Biotinylated MR1-β2m-Ag was immobilized on SA-Chip (GE) with a surface density of ~2,000 response units (RU). Various concentrations (0–200 µM) of three cord blood TCRs, CB946 A1 (TRAV26-2/TRBV4-1b), CB946 A2 (TRAV1-2/TRBV6-2) and CB946 A4 (TRAV5/TRBV12-3), as well as two adult MAIT TCRs; A-F7 (TRAV1-2-TRBV6-1) and #6 (TRAV1-2-TRBV6-4) were injected over the bound MR1-β2m-Ag at 5 µL/min and equilibrium data were collected. The final response was calculated by subtracting the response of the blank flow cell alone from the TCR-MR1-β2m-Ag complex. The SPR sensograms, equilibrium curves and steady state K_D_ values (µM) were prepared using GraphPad Prism 10.

### Crystallization, data collection, structure determination, and analysis

Purified CB964 A2 TCR was mixed with MR1-β2m-5-OP-RU in a 1:1 molar ratio and then held on ice for 2 h. The concentration of the mixture was 4–6 mg/ml. CB964 A2 TCR-MR1-5-OP-RU crystals were created at 20 °C using the hanging-drop vapor diffusion method^38^. The precipitant reservoir solution consisted of 100 mM Bis–Tris Propane (BTP; pH 6.0–6.7), 10–20% PEG3350, and 200 mM sodium acetate. The ternary complex crystals were grown for 6 weeks and harvested before being quickly soaked in reservoir solution containing 15% glycerol for cryoprotection and then flash-frozen in liquid nitrogen. At the Australian Synchrotron, X-ray diffraction data sets were collected at 100 K using the MX2 beamline. Diffraction data were processed using XDS^61^ and programs from the CCP4 suite^62^ and Phenix package^63^. The ternary structure of CB964 A2 TCR-MR1-5-OP-RU was determined by molecular replacement using PHASER^64^, where modified TCR-MR1 ternary complex (PDB: 6PUC) was used as a search model. After building the models in COOT^65^ and refining them iteratively with Phenix.refine^63^, the models were validated with MolProbity^66^. Supplemental Table 2 provides a summary of CB964 A2 TCR-MR1-5-OP-RU final crystallographic data. BSA calculations were performed using PISA^67^ and the contacts generated by the Contact program from the CCP4 suite^62^. Molecular graphics visualization was generated using PyMOL v2.5 (Schrödinger).

### Statistics

Additional analyses based on sex were not performed given the small sample size and unknown sex of the neonates from whom the cord blood samples were obtained. Statistical analysis was done in R using built-in statistical packages for all differential gene expression of single-cell gene expression data. Given the possibility of increased false discoveries with single-cell data due to mean expression bias^68^, we transformed our single-cell gene expression data into pseudobulked gene expression prior to performing differential gene expression using the edgeR package^69^. The only exception to this, was the analysis of CB participants’ TRAV1-2( + ) MAIT cells compared to TRAV1-2(-) MR1T cells given the lower cell counts for this analysis. For this analysis we used a Wilcoxon test for statistical significance. For all analysis we considered a 2-log threshold change of more than 0.5 and a false discovery rate (FDR) or Bonferroni-corrected *p*-value of <0.05 to be significant. For TCR analysis, data was first analyzed in R studio and then diversity and pairing preference data was extracted and analyzed in Prism. Statistics were performed in Prism using a *t*-test for significance, with *p* < 0.05 being considered significant.

### Reporting summary

Further information on research design is available in the Nature Portfolio Reporting Summary linked to this article.

## Supplementary information

Supplemental Information

Description of Additional Supplementary Files

Supplemental Data 1

Supplemental Data 2

Supplemental Data 3

Supplemental Data 4

Supplemental Data 5

Reporting Summary

Transparent Peer Review file

## Source data

Source Data for Figure 9

Source Data

## Data Availability

All raw data have been deposited to dbgap under the accession number: phs003810.v1.p1. The authors declare that the data supporting the findings of this study are available within the paper and its supplementary information files. The source data underlying the graphs in the paper can be found in the Supplementary Data. The accession number for the atomic coordinates of CB964 A2 TCR-MR1-5-OP-RU, along with associated structure factors, have been deposited at the protein databank (www.rcsb.org) with accession code 9MS0. All data are included in the Supplementary Information or available from the authors, as are unique reagents used in this Article. The raw numbers for charts and graphs are available in the Source Data file whenever possible. Source data are provided with this paper.

## Code availability

All R studio code used in the generation of this analysis is freely available and uploaded to github (https://github.com/kaindylan/Cordblood-Paper).
