# Supplementary material for: Human neonatal MR1T cells have more diverse TCR repertoires but reduced bacterial recognition than adult MR1T cells
Source: Nat Commun. 2026 Jun 29;17:8127. doi: 10.1038/s41467-026-74998-7 (PMC13458692; doi:10.1038/s41467-026-74998-7)
Supplement: Supplementary file 1 [file 41467_2026_74998_MOESM1_ESM.pdf]

Supplementary Information

# Human neonatal MR1T cells have more diverse TCR repertoires but reduced bacterial recognition than adult MR1T cells pathogens

Dylan Kain*, Wael Awad*, GW McElfresh, Meghan Cansler, Gwendolyn M. Swarbrick, Kean Chan Yew Poa, Conor McNeice, Gregory Boggy, Katherine H. Rott, Megan D. Null, David M. Lewinsohn, Jamie Rossjohn, Benjamin N. Bimber, Deborah A. Lewinsohn#

*These authors contributed equally

# Corresponding author. Email: lewinsde@ohsu.edu

**Contents:**

Supplementary Figs. 1-10

Supplementary Tables 1- 5

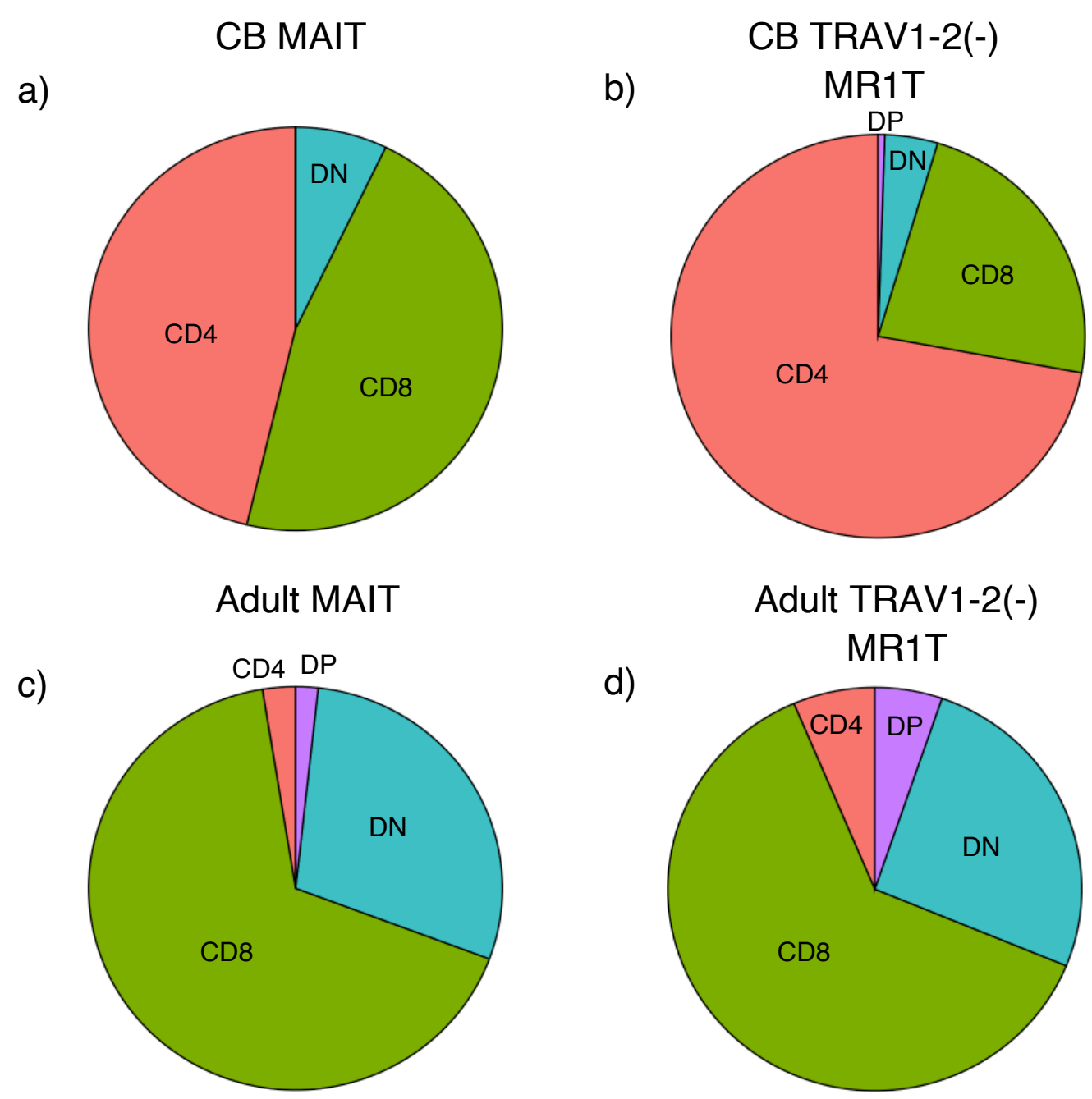


**Supplemental Fig. 1 CD4 and CD8 protein expression.**

a,b) Cord blood (CB) MR1-restricted T (MR1T) cell expression of CD8 and CD4 based on CITE-seq cell surface expression for a) mucosal associated invariant T (MAIT) cells or b) TRAV 1-2(-) cells, based on single cell TCR sequencing (scTCR-seq). c,d) Adult MR1T cell expression of CD8 and CD4 based on CITE-seq cell surface expression for c) MAIT cells or d)TRAV 1-2(-) cells based on scTCR-seq.

DP = double positive, DN = double negative.

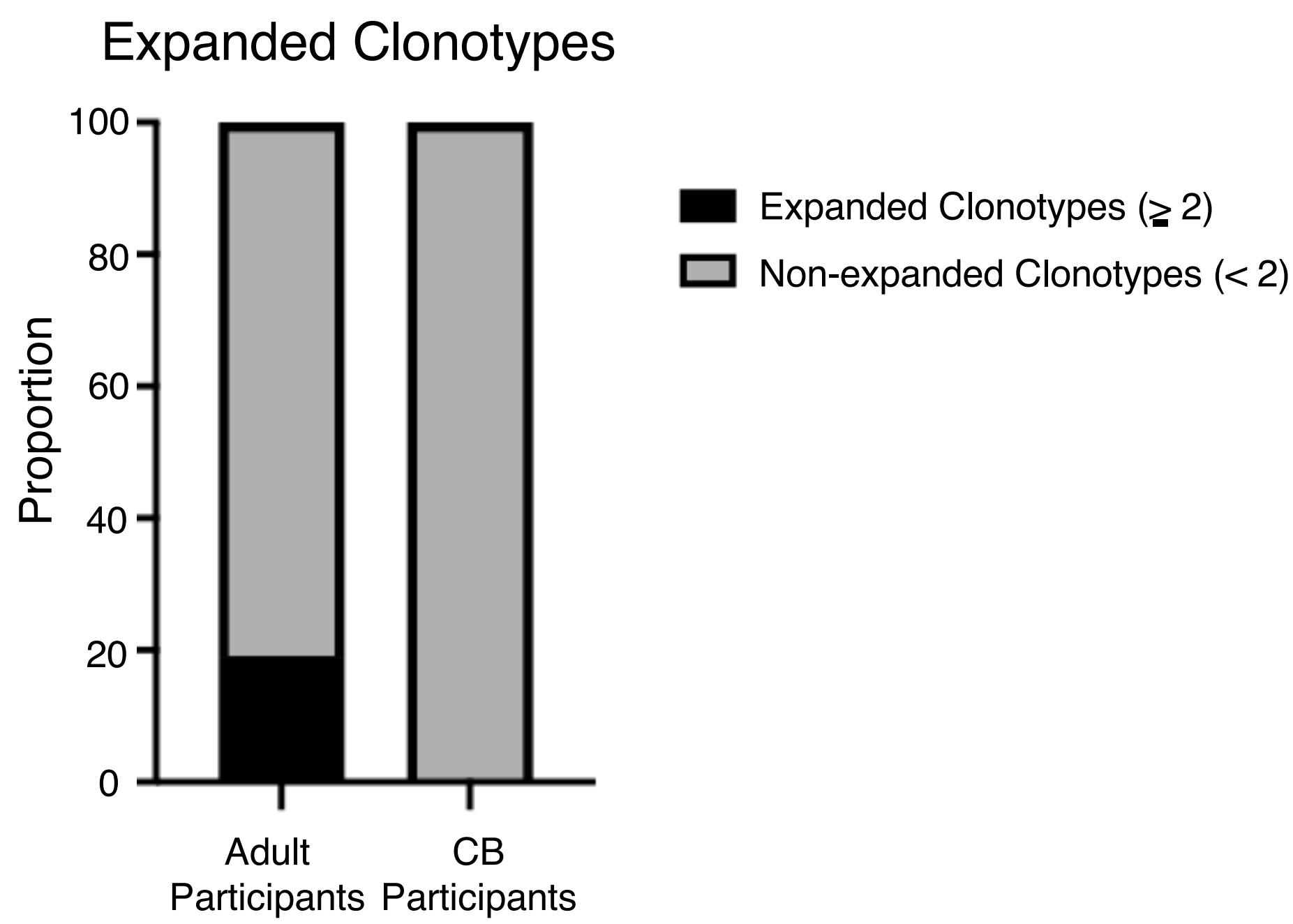


**Supplemental Fig. 2 Proportion of expanded clones for cord blood and adult MR1T cells.**

Proportion of MR1-restricted (MR1T) cells from adult or cord blood (CB) participants with at least one other cell with an identical CDR3.

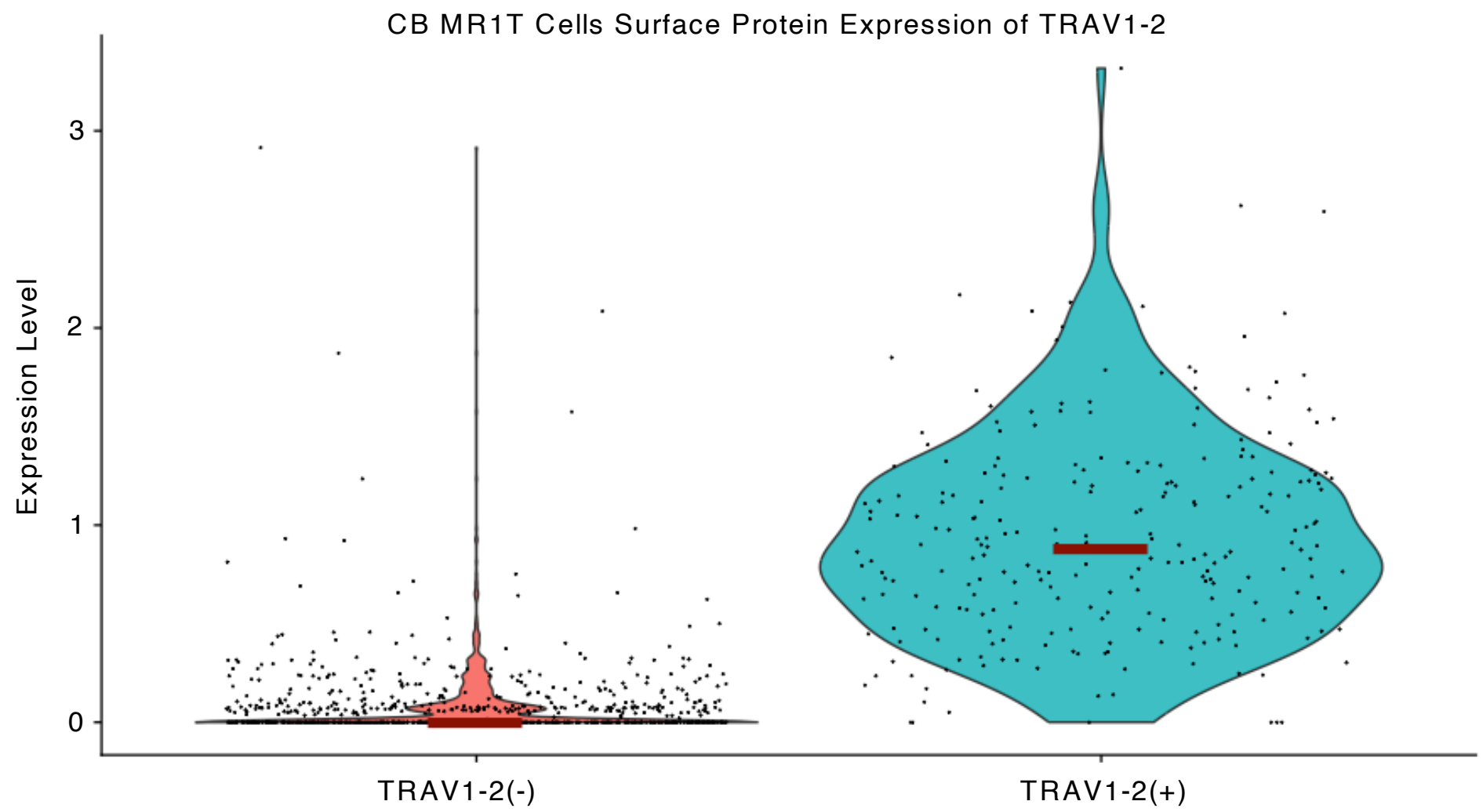


**Supplemental Fig. 3 TRAV1-2 cell surface protein expression matches TRAV1-2 gene expression for CB MR1T cells.**

Cord blood (CB) MR1-restricted T (MR1T) cells were sorted into TRAV1-2(+) or TRAV1-2(-) based on gene expression, and violin plot of cell surface protein expression is displayed based on CITE-seq staining for TRAV1-2 (TCR Vα7.2).

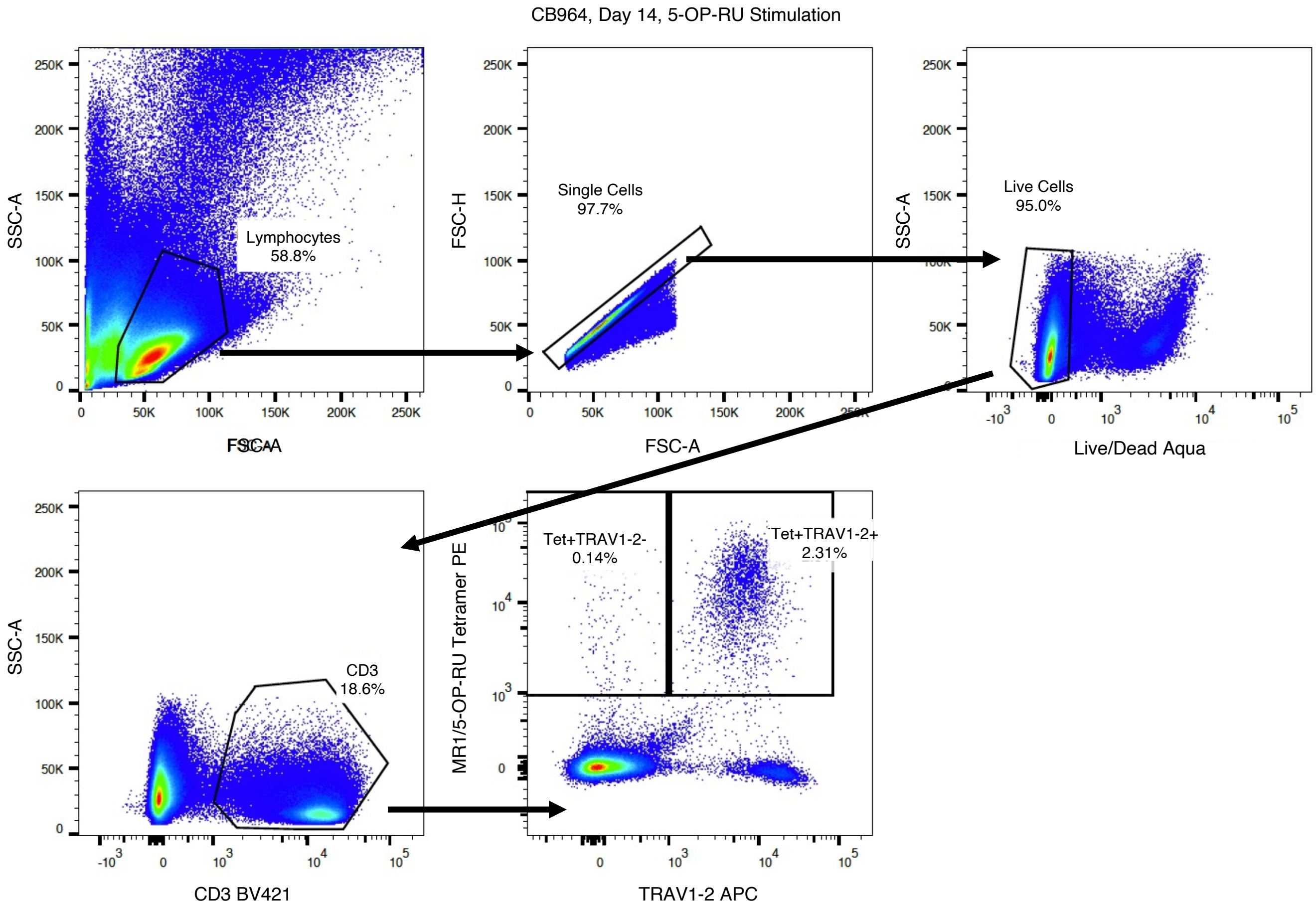


**Supplemental Fig. 4 Representative gating strategy for 5-OP-RU stimulation experiment.** Representative gating strategy for the 5-OP-RU stimulation experiment shown in Figure 4.

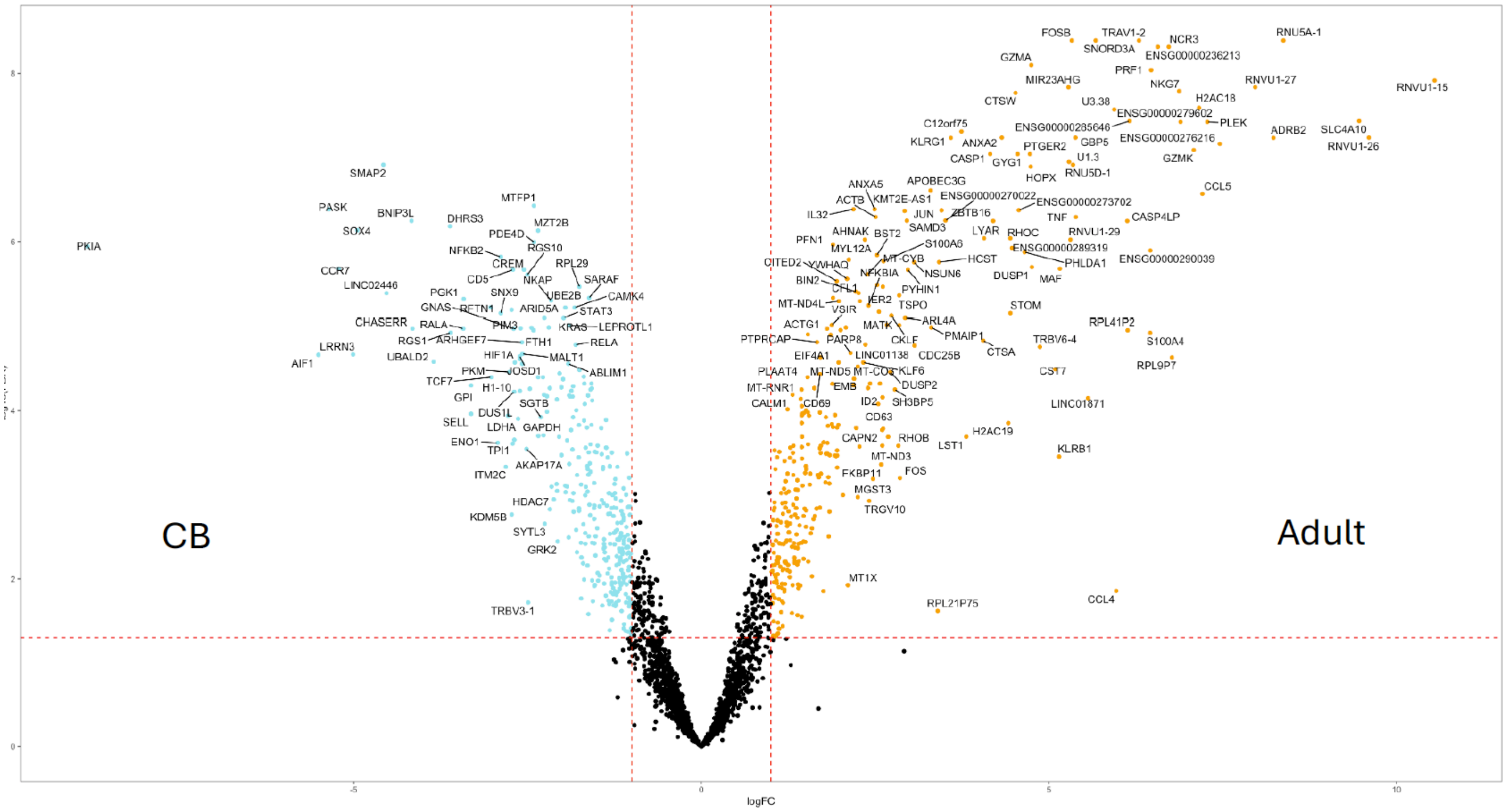


**Supplemental Figure 5. Volcano plot for differential gene expression of CB TRAV1-2(-) MR1T cells vs Adult TRAV1-2(-) MR1T cells.**

Labeled genes were greater than 0.5 or less than -0.5 log fold change and had a Bonferroni-adjusted p-value of < 0.05.

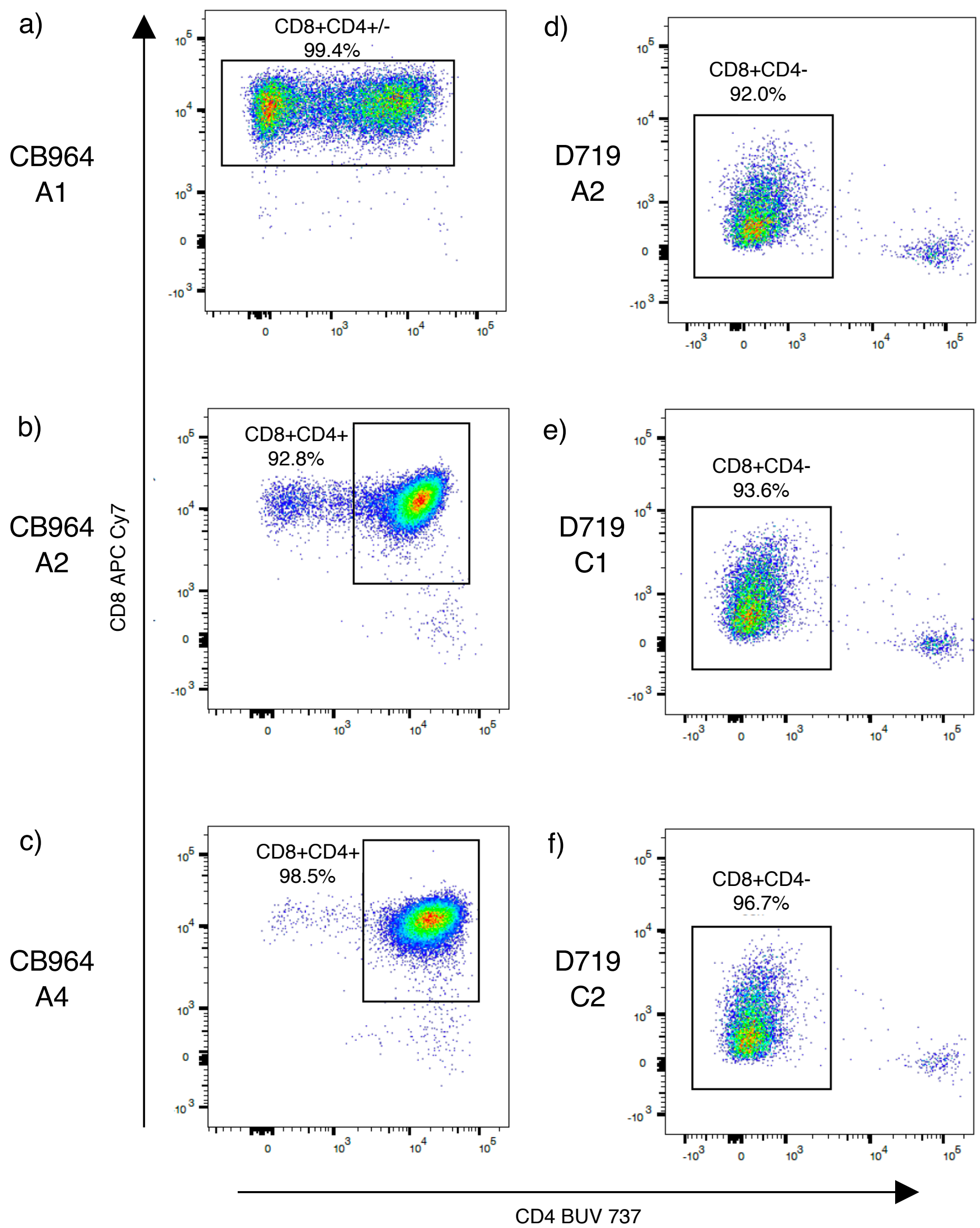


**Supplemental Fig. 6 CD8 and CD4 expression on CB and adult MR1T cell clones.**

a-c) CD4 and CD8 staining of cord blood (CB) MR1-restricted T (MR1T) cell clones and analysis by flow cytometry. d-f) CD4 and CD8 staining of adult MR1T cell clones and analysis by flow cytometry.

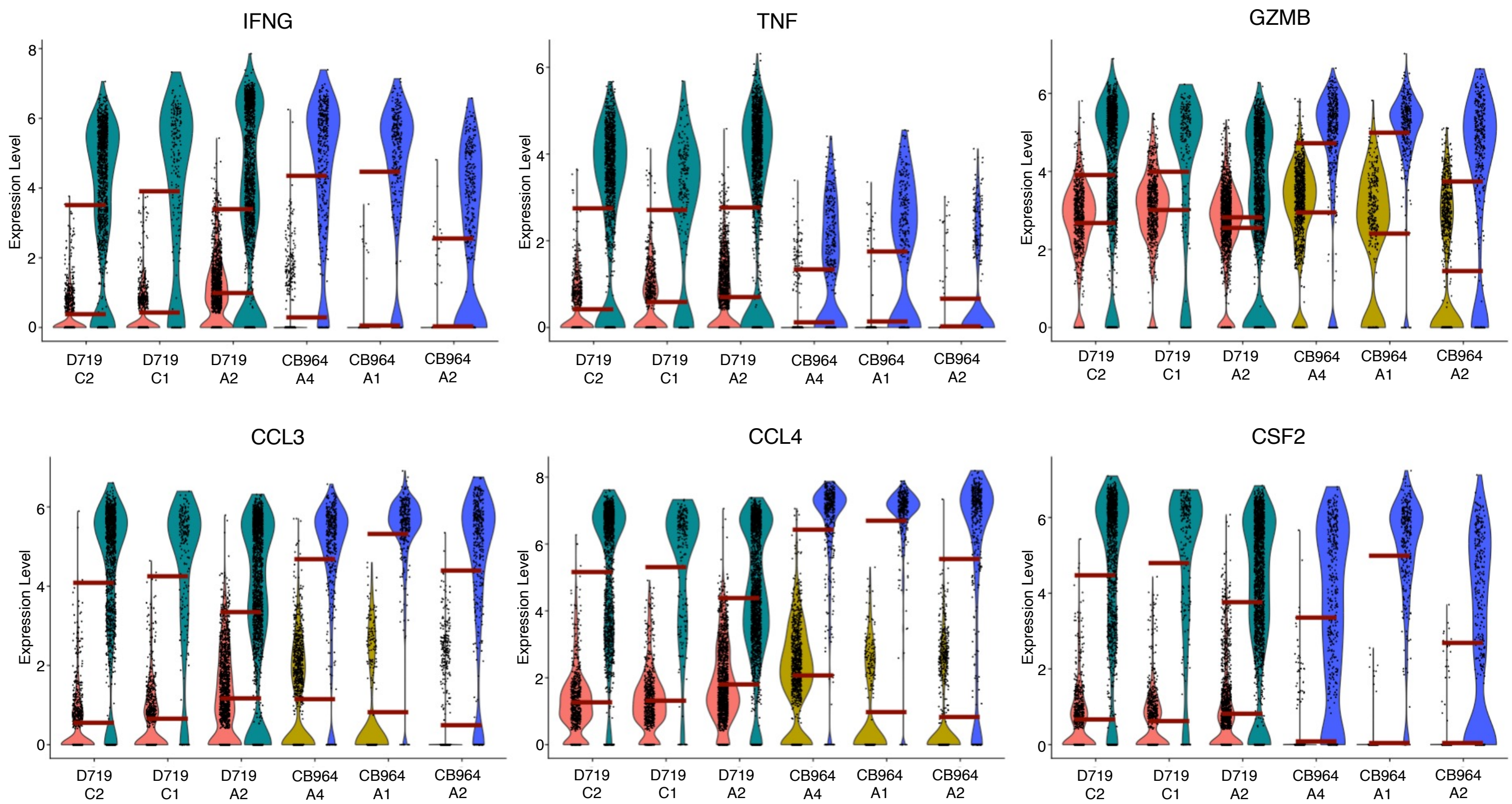


**Supplemental Fig. 7 Individual CB MR1T cell clones produce typical inflammatory/cytotoxic cytokines by gene expression.**

Violin plot of gene expression of common inflammatory/cytotoxic genes from stimulated (PMA/Ionomycin) and unstimulated MR1-restricted T (MR1T) cell clones comparing clones isolated from an adult (D719) to those isolated from cord blood (CB; CB964) broken out by each individual clone. IFNG = IFN-γ; GZMB = granzyme B.

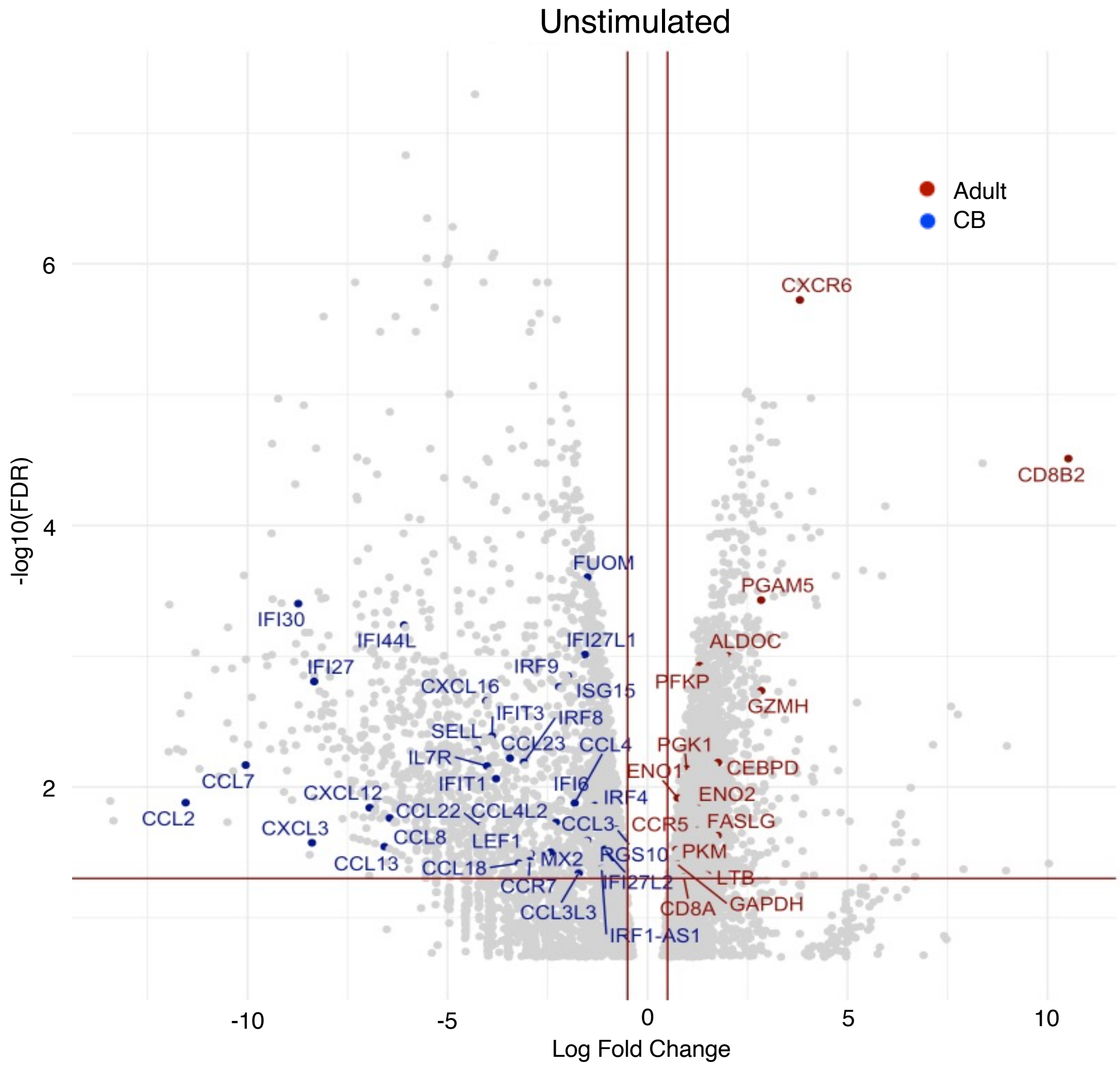


**Supplemental Fig. 8 Differential gene expression of unstimulated CB and adult MR1T cell clones.**

Volcano plot of differentially expressed genes of unstimulated adult compared to unstimulated cord blood (CB) MR1-restricted T (MR1T) cell clones. The p-values were calculated using the Wilcoxon signed-rank test with Bonferroni adjustment for multiple comparisons. Horizontal red line represents Bonferroni-adjusted p-value of 0.05, and vertical red lines represent log fold change of 0.5 or -0.5.

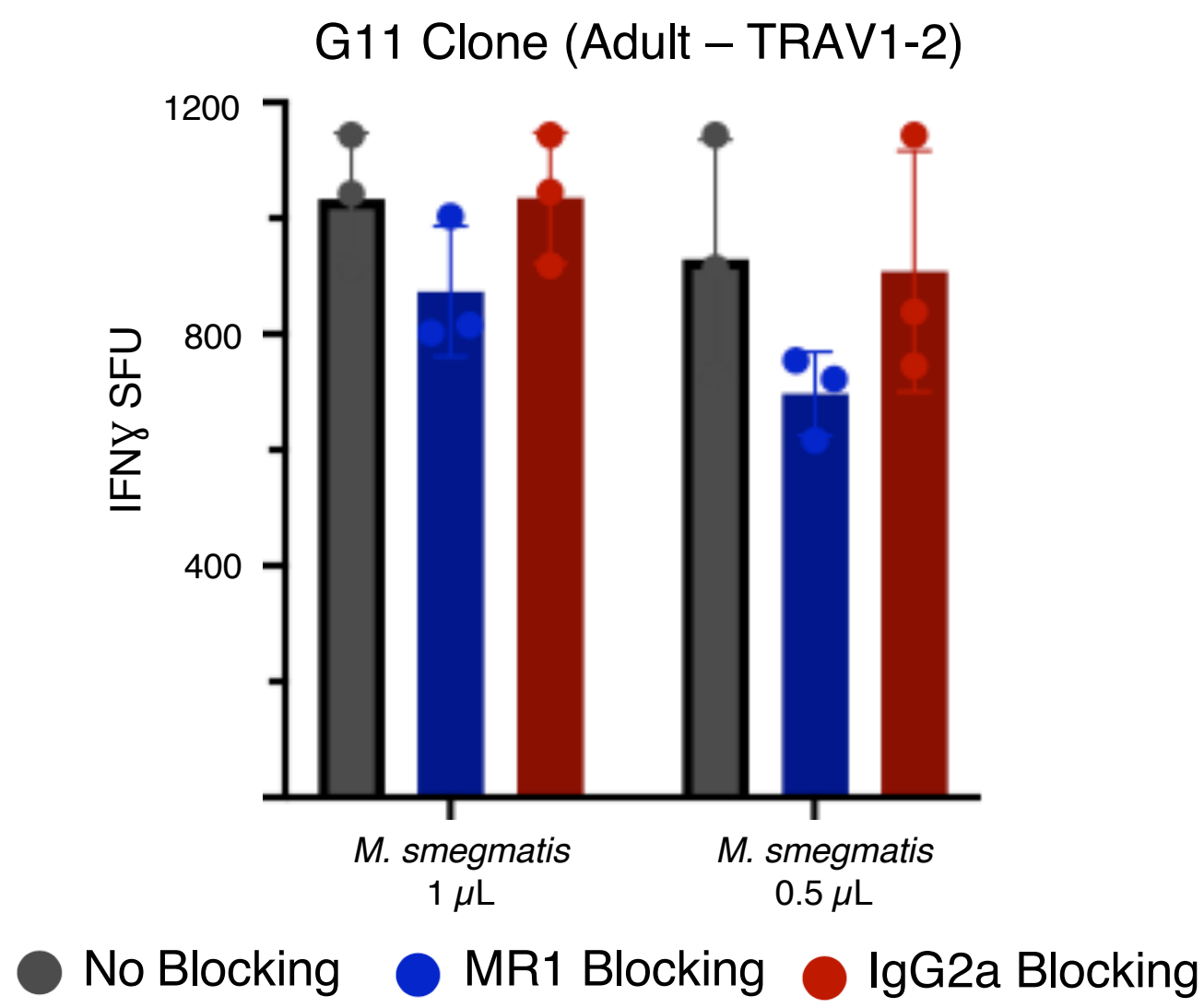


**Supplemental Fig. 9 Titration of *Mycobacterium smegmatis*.**
IFNγ ELISPOT of adult MR1-restriceted (MR1T) cell clone (G11) with lower concentrations of *M. smegmatis* than shown in Fig. 8h. Responses without blocking, anti-MR1 blocking antibody or isotype control blocking antibody are represented. Error bars represent the standard deviation from 3 biological replicates.

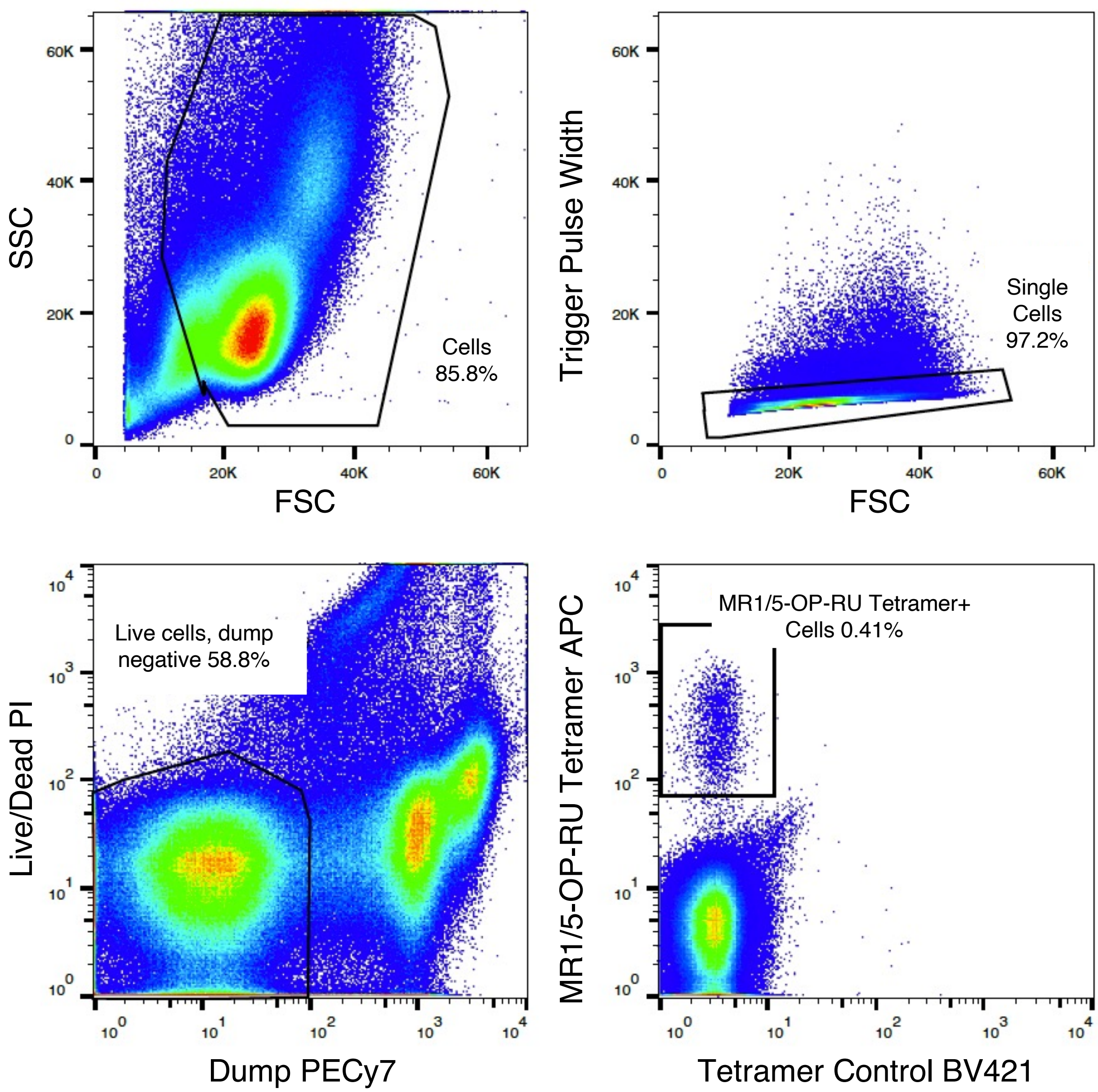


**Supplemental Fig. 10 Representative FACS sorting strategy.**
Live MR1/5-OP-RU positive cells were sorted based on PI negativity, negative staining (dump channel) for CD14, CD15, CD19, CD123, CD235ab (PeCy7), and MR1/5-OP-RU tetramer positive. MR1T cells sorted in this manner were used for the data shown in Figures 1-3 & 5.

**Supplemental Table 1** Diffraction data collection and refinement statistics

| | Cord A2 TCR-MR1-5-OP-RU |
|---|---|
| Wavelength (Å) | 0.9537 |
| Resolution range (Å) | 46.84 - 2.52 (2.59 - 2.52) |
| Space group | P 21 21 21 |
| Unit cell<br>a, b, c (Å)<br>α, β, γ (°) | 62.526 114.533 141.3<br><br>90 90 90 |
| Total reflections | 69666 (5720) |
| Unique reflections | 34953 (2871) |
| Multiplicity | 2.0 (2.0) |
| Completeness (%) | 99.57 (99.65) |
| Mean I/sigma(I) | 10.20 (2.04) |
| Wilson B-factor | 41.61 |
| R-merge | 0.0467 (0.359) |
| R-meas | 0.06605 (0.5077) |
| R-pim | 0.0467 (0.359) |
| CC1/2 | 0.997 (0.768) |
| CC* | 0.999 (0.932) |
| R-work | 0.1827 (0.2765) |
| R-free | 0.2087 (0.3104) |
| Number of non-hydrogen atoms | 6708 |
| macromolecules | 6314 |
| ligands | 22 |
| solvent | 372 |
| Protein residues | 791 |
| RMSD (bonds) (Å) | 0.116 |
| RMSD (angles) (°) | 2.37 |
| Ramachandran favored (%) | 97.29 |
| Ramachandran allowed (%) | 2.58 |
| Ramachandran outliers (%) | 0.13 |
| Rotamer outliers (%) | 4.33 |
| Average B-factor | 47.41 |
| macromolecules | 47.46 |
| ligands | 28.16 |
| solvent | 47.65 |

Statistics for the highest-resolution shell are shown in parentheses.

**Supplemental Table 2** Contacts of A2 TCR with MR1-5-OP-RU

| TCR gene | TCR residues | MR1 residues | Bond type |
|---|---|---|---|
| CDR1α | Gly$^{28}$ | Glu$^{160}$ | VDW |
| | Phe$^{29}$ | Glu$^{160}$ | VDW |
| | Phe$^{29\text{-N}}$ | Glu$^{160\text{-O}\varepsilon 2}$ | H-bond |
| | Phe$^{29\text{-O}}$ | Asn$^{155\text{-N}\delta 2}$, Glu$^{160\text{-O}\varepsilon 2}$ | H-bond |
| | Asn$^{30}$ | Tyr$^{152}$, Trp$^{156}$, Glu$^{160}$ | VDW |
| CDR2α | Tyr$^{48}$ | His$^{148}$, Tyr$^{152}$ | VDW |
| | Val$^{50}$ | Leu$^{151}$, Tyr$^{152}$, Asn$^{155}$ | VDW |
| | Leu$^{51}$ | Leu$^{151}$, Lys$^{154}$ | VDW |
| | Glu$^{55}$ | His$^{148}$ | VDW |
| FWα | Arg$^{66}$ | Asn$^{155}$, Glu$^{159}$ | VDW |
| | Arg$^{66\text{-N}\eta 1}$ | Asn$^{155\text{-O}\delta 1}$ | H-bond |
| CDR3α | Ser$^{93}$ | Tyr$^{62}$, Glu$^{160}$, Trp$^{164}$ | VDW |
| | Ser$^{93\text{-O}}$ | Tyr$^{62\text{-OH}}$ | H-bond |
| | Ser$^{94\text{-O}}$ | Arg$^{61\text{-N}\varepsilon}$, Arg$^{61\text{-N}\eta 2}$ | H-bond |
| | Ser$^{94}$ | Arg$^{61}$, Leu$^{65}$ | VDW |
| | Tyr$^{95}$ | Arg$^{61}$, Tyr$^{62}$, Leu$^{65}$, Tyr$^{152}$, Trp$^{156}$ | VDW |
| | Tyr$^{95\text{-O}}$ | Arg$^{61\text{-N}\eta 2}$ | H-bond |
| | Tyr$^{95\text{-OH}}$ | Tyr$^{152\text{-OH}}$, Trp$^{156\text{-N}\varepsilon 1}$ | H-bond |
| CDR1β | Tyr$^{31}$ | Gly$^{68}$ | VDW |
| CDR2β | Tyr$^{48\text{-OH}}$ | Arg$^{61\text{-N}\eta 1}$ | H-bond |
| | Tyr$^{48}$ | Arg$^{61}$, Gln$^{64}$ | VDW |
| | Val$^{50}$ | Gln$^{64}$ | VDW |
| FWβ | Ala$^{56}$ | Gln$^{64}$ | VDW |
| CDR3β | Thr$^{97\text{-O}\gamma 1}$ | Trp$^{69\text{-N}\varepsilon 1}$ | H-bond |
| | Thr$^{97}$ | Leu$^{65}$ | VDW |
| | Asn$^{98\text{-O}\delta 1}$ | Glu$^{149\text{-O}\varepsilon 2}$ | H-bond |
| | Asn$^{98\text{-N}\delta 2}$ | Glu$^{149\text{-O}\varepsilon 2}$ | H-bond |
| | Asn$^{98}$ | Glu$^{149}$, Tyr$^{152}$ | VDW |
| | Tyr$^{99\text{-OH}}$ | His$^{148\text{-N}\delta 1}$ | H-bond |
| | Tyr$^{99}$ | His$^{148}$ | VDW |

- Atomic contacts determined using the *CONTACT* program of the CCP4i package with cutoff of 4 Å.
- Hydrogen bond interactions are defined as contact distances of 3.5 Å or less.
- Van der Waals (VDW) interactions are defined as non-hydrogen bond contact distances of less than 4 Å.
- R: 5-OP-RU

Supplemental Table 3: Reagents used for FACS sorting

| Reagent | CAT | Company | Dilution used |
|---|---|---|---|
| CD14 PeCy7 | 301814 | Biolegend | 1:25 |
| CD15 PeCy7 | 301924 | Biolegend | 1:10 |
| CD19 PeCy7 | 302216 | Biolegend | 1:25 |
| CD123 PeCy7 | 306010 | Biolegend | 1:17 |
| CD235ab PeCy7 | 306620 | Biolegend | 1:50 |
| Proprium Iodide Solution | 130-093-233 | Miltenyi Biotec | 1:800 |
| MR1/5-OP-RU Tetramer PE | NA | NIH Tetramer Core | 1:500 for adults<br>1:250 for Cord |

Supplemental Table 4: Reagents used for 10X Genomics Staining

| Reagent | CAT | Company | Dilution used |
|---|---|---|---|
| TotalSeq Hashtag 1 | 394661 | Biolegend | 1:200 |
| TotalSeq Hashtag 2 | 394663 | Biolegend | 1:200 |
| TotalSeq Hashtag 3 | 394665 | Biolegend | 1:200 |
| TotalSeq Hashtag 4 | 394667 | Biolegend | 1:200 |
| TotalSeq Hashtag 5 | 394669 | Biolegend | 1:200 |
| TotalSeq CD4 | 300567 | Biolegend | 1:500 |
| TotalSeq CD8 | 344753 | Biolegend | 1:500 |
| TotalSeq TCR Vα7.2 | 351735 | Biolegend | 1:500 |
| TotalSeq CD26 | 302722 | Biolegend | 1:500 |
| TotalSeq CD161 | 339947 | Biolegend | 1:500 |
| TotalSeq CD197 (CCR7) | 353251 | Biolegend | 1:200 |
| TotalSeq CD279 (PD-1) | 329963 | Biolegend | 1:200 |
| TotalSeq CD45RA | 304163 | Biolegend | 1:500 |
| Human TruStain FcX | 422302 | Biolegend | 1:25 |

Supplemental Table 5: Reagents used for MR1T cell clone and CBMC stimulation flow cytometry

| Reagent | CAT | Company | Dilution used |
|---|---|---|---|
| CD8 APC Cy7 | 344714 | Biolegend | 1:500 |
| CD3 BV421 | 317344 | Biolegend | 1:25 |
| CD4 BUV737 | 612748 | BD Horizon | 1:100 |
| CD26 FITC | 302704 | Biolegend | 1:50 |
| CD161 PeCy7 | 339918 | Biolegend | 1:50 |
| TRAV1-2 PerCPCy55 | 351710 | Biolegend | 1:25 |
| Live Dead Aqua | L34966 | Thermo Fischer Scientific | 1:200 |
| MR1/5-OP-RU Tetramer PE | NA | NIH Tetramer Core | 1:500 |
