# Supplementary material for: Human neonatal MR1T cells have more diverse TCR repertoires but reduced bacterial recognition than adult MR1T cells
Source: Nat Commun. 2026 Jun 29;17:8127. doi: 10.1038/s41467-026-74998-7 (PMC13458692; doi:10.1038/s41467-026-74998-7)
Supplement: Supplementary file 2 [file 41467_2026_74998_MOESM2_ESM.pdf]

Description of Additional Supplementary Files

Supplemental Data 1: Differentially expressed genes between cord blood and adult MR1T cells. Statistical significance calculated using Wilcoxon signed-rank test with Bonferroni correction for multiple comparisons.

Supplemental Data 2: Differentially expressed genes between cord blood TRAV1-2(+) and TRAV1-2(-) MR1T cells. Statistical significance calculated using Wilcoxon signed-rank test with Bonferroni correction for multiple comparisons.

Supplemental Data 3: Differentially expressed genes between cord blood and adult TRAV1-2(+) MAIT cells. Statistical significance calculated using Wilcoxon signed-rank test with Bonferroni correction for multiple comparisons.

Supplemental Data 4: Differentially expressed genes between cord blood and adult unstimulated MR1T cell clones. Statistical significance calculated using Wilcoxon signed-rank test with Bonferroni correction for multiple comparisons.

Supplemental Data 5: Differentially expressed genes between cord blood and adult stimulated MR1T cell clones. Statistical significance calculated using Wilcoxon signed-rank test with Bonferroni correction for multiple comparisons.
