# Supplementary material for: Human neonatal MR1T cells have more diverse TCR repertoires but reduced bacterial recognition than adult MR1T cells
Source: Nat Commun. 2026 Jun 29;17:8127. doi: 10.1038/s41467-026-74998-7 (PMC13458692; doi:10.1038/s41467-026-74998-7)
Supplement: Supplementary file 3 [file 41467_2026_74998_MOESM8_ESM.pdf]

nature portfolio

Corresponding author(s): Deborah Lewinsohn

Last updated by author(s): Mar 28, 2025

Reporting Summary

Nature Portfolio wishes to improve the reproducibility of the work that we publish. This form provides structure for consistency and transparency in reporting. For further information on Nature Portfolio policies, see our Editorial Policies and the Editorial Policy Checklist.

Statistics

For all statistical analyses, confirm that the following items are present in the figure legend, table legend, main text, or Methods section.

n/a	Confirmed	
[ ]	[x]	The exact sample size (*n*) for each experimental group/condition, given as a discrete number and unit of measurement
[ ]	[x]	A statement on whether measurements were taken from distinct samples or whether the same sample was measured repeatedly
[ ]	[x]	The statistical test(s) used AND whether they are one- or two-sided<br>*Only common tests should be described solely by name; describe more complex techniques in the Methods section.*
[ ]	[x]	A description of all covariates tested
[ ]	[x]	A description of any assumptions or corrections, such as tests of normality and adjustment for multiple comparisons
[ ]	[x]	A full description of the statistical parameters including central tendency (e.g. means) or other basic estimates (e.g. regression coefficient) AND variation (e.g. standard deviation) or associated estimates of uncertainty (e.g. confidence intervals)
[ ]	[x]	For null hypothesis testing, the test statistic (e.g. F, t, r) with confidence intervals, effect sizes, degrees of freedom and P value noted<br>*Give P values as exact values whenever suitable.*
[x]	[ ]	For Bayesian analysis, information on the choice of priors and Markov chain Monte Carlo settings
[x]	[ ]	For hierarchical and complex designs, identification of the appropriate level for tests and full reporting of outcomes
[x]	[ ]	Estimates of effect sizes (e.g. Cohen's *d*, Pearson's *r*), indicating how they were calculated

Our web collection on statistics for biologists contains articles on many of the points above.

Software and code

Policy information about availability of computer code

Data collection	FACS diva, Elispot AID software, cell ranger
Data analysis	https://github.com/kaindylan/Cordblood-Paper/blob/main/Cordblood_paper_clean_markdown.Rmd<br>Graphpad prism version 10<br>Flowjo version 10.7.1

For manuscripts utilizing custom algorithms or software that are central to the research but not yet described in published literature, software must be made available to editors and reviewers. We strongly encourage code deposition in a community repository (e.g. GitHub). See the Nature Portfolio guidelines for submitting code & software for further information.

Data

Policy information about availability of data

All manuscripts must include a data availability statement. This statement should provide the following information, where applicable:

- Accession codes, unique identifiers, or web links for publicly available datasets
- A description of any restrictions on data availability
- For clinical datasets or third party data, please ensure that the statement adheres to our policy

The data that support the findings of this study are not openly available due to reasons of sensitivity and are available from the corresponding author upon reasonable request. Data are located in controlled access data storage at dbGaP (accessions number: phs003810.v1.p1)

Research involving human participants, their data, or biological material

Policy information about studies with human participants or human data. See also policy information about sex, gender (identity/presentation), and sexual orientation and race, ethnicity and racism.

Reporting on sex and gender	Adult participants were mixed male (n = 2) and female (n = 3). Gender was not reported for cord blood samples as the participants are neonates and de-identified prior to access to these samples and are thus unknown. Additional testing based on sex was not done given small sample size.
Reporting on race, ethnicity, or other socially relevant groupings	Cord blood samples are de-identified and thus we do not have data on race, ethnicity or other socially relevant groupings. All adult participants were caucasian. Additional subgroup testing was not not done given small sample size.
Population characteristics	These are described with the main important feature being age, which is the basis for the study.
Recruitment	Adult participants were recruits through fliers at the university campus. Cord blood samples were obtained from OHSU deliveries through an IRB exempt protocol.
Ethics oversight	Ethics was approved by the Institutional Review Board at Oregon Health & Science University (OHSU; IRB00000186 and IRB#5032)

Note that full information on the approval of the study protocol must also be provided in the manuscript.

Field-specific reporting

Please select the one below that is the best fit for your research. If you are not sure, read the appropriate sections before making your selection.

[x] Life sciences [ ] Behavioural & social sciences [ ] Ecological, evolutionary & environmental sciences

For a reference copy of the document with all sections, see nature.com/documents/nr-reporting-summary-flat.pdf

Life sciences study design

All studies must disclose on these points even when the disclosure is negative.

Sample size	Based on 5 donors per group, and recovery of approximately 300-400 cells per cord blood donor (based on pilot testing), this would mean that 80% power would be met with an effect size around 1.3-1.5
Data exclusions	All adult participants needed to be healthy. All cord blood participants needed to have been healthy term births.
Replication	All data were successful replicated the number of times disclosed in figure legend.
Randomization	Not applicable since the main variable was age which cannot be randomized.
Blinding	Blinding was not possible for this study as the main variable being examined is age, and there are easily identifiable differences at all steps of the study that would make blinding impossible. For example, at birth there are far fewer MR1T cells and as a results at the time of FACS sorting it would be easy to identify the differences. At the analysis level, there are phenotypic differences that would make determining the participant age group very apparent.

Reporting for specific materials, systems and methods

We require information from authors about some types of materials, experimental systems and methods used in many studies. Here, indicate whether each material, system or method listed is relevant to your study. If you are not sure if a list item applies to your research, read the appropriate section before selecting a response.

Materials & experimental systems

n/a	Involved in the study
[ ]	[x] Antibodies
[ ]	[x] Eukaryotic cell lines
[x]	[ ] Palaeontology and archaeology
[x]	[ ] Animals and other organisms
[x]	[ ] Clinical data
[x]	[ ] Dual use research of concern
[x]	[ ] Plants

Methods

n/a	Involved in the study
[x]	[ ] ChIP-seq
[ ]	[x] Flow cytometry
[x]	[ ] MRI-based neuroimaging

Antibodies

Antibodies used

CD14 PeCy7 301814 Biolegend
CD15 PeCy7 301924 Biolegend
CD19 PeCy7 302216 Biolegend
CD123 PeCy7 306010 Biolegend
CD235ab PeCy7 306620 Biolegend
Proprium Iodide Solution 130-093-233 Miltenyi Biotec
TotalSeq Hashtag 1 394661 Biolegend
TotalSeq Hashtag 2 394663 Biolegend
TotalSeq Hashtag 3 394665 Biolegend
TotalSeq Hashtag 4 394667 Biolegend
TotalSeq Hashtag 5 394669 Biolegend
TotalSeq CD4 300567 Biolegend
TotalSeq CD8 344753 Biolegend
TotalSeq TCR V7.2 351735 Biolegend
TotalSeq CD26 302722 Biolegend
TotalSeq CD161 339947 Biolegend
TotalSeq CD197 (CCR7) 353251 Biolegend
TotalSeq CD279 (PD-1) 329963 Biolegend
TotalSeq CD45RA 304163 Biolegend
Human TruStain FcX 422302 Biolegend
CD8 APC Cy7 344714 Biolegend
CD3 BV421 317344 Biolegend
CD4 BUV737 612748 BD Horizon
CD26 FITC 302704 Biolegend
CD161 PeCy7 339918 Biolegend
TRAV1-2 PerCPCy55 351710 Biolegend
Live Dead Aqua L34966 Thermo Fischer Scientific

Validation

Each primary antibody was titrated using

Eukaryotic cell lines

Policy information about cell lines and Sex and Gender in Research

Cell line source(s)

Human PBMC (Dendritic cells, B-LCL, T cell clones). D426G11 T cell clone, sex male. D719A2 sex female, D719C1 sex female, D719C2 sex female, CB964A1 sex unknown, CB964A2 sex unknown, CB964A4 sex unknown, DC donor sex male.

Authentication

T cell clones are expanded from frozen stocks using published protocols and are used for 2 weeks and then discarded. They are also tested to confirm their phenotype using either ELISPOT or flow cytometry. BEAS-2B cells were bought from ATCC. These cell lines are passaged up to 15 times before being discarded. Freezebacks are frozen down during the first 2-3 passages.

Mycoplasma contamination

All cells were tested for mycoplasma and tested negative

Commonly misidentified lines
(See ICLAC register)

N/A

Plants

Seed stocks	N/A
Novel plant genotypes	N/A
Authentication	N/A

Flow Cytometry

Plots

Confirm that:

- [x] The axis labels state the marker and fluorochrome used (e.g. CD4-FITC).
- [x] The axis scales are clearly visible. Include numbers along axes only for bottom left plot of group (a 'group' is an analysis of identical markers).
- [x] All plots are contour plots with outliers or pseudocolor plots.
- [x] A numerical value for number of cells or percentage (with statistics) is provided.

Methodology

Sample preparation	This is described in detail for each assay in the methods.
Instrument	BD FACS symphony
Software	Acquisition on FACS Diva. Analysis with Flowjo
Cell population abundance	Purity check was done after each sort and was > 95%
Gating strategy	Gating strategy shown in supplemental figure

- [x] Tick this box to confirm that a figure exemplifying the gating strategy is provided in the Supplementary Information.
