# Supplementary material for: Human neonatal MR1T cells have more diverse TCR repertoires but reduced bacterial recognition than adult MR1T cells
Source: Nat Commun. 2026 Jun 29;17:8127. doi: 10.1038/s41467-026-74998-7 (PMC13458692; doi:10.1038/s41467-026-74998-7)
Supplement: Supplementary file 4 [file 41467_2026_74998_MOESM9_ESM.pdf]

nature portfolio

Peer Review File

Human neonatal MR1T cells have more diverse TCR repertoires but reduced bacterial recognition than adult MR1T cells

Corresponding Author: Dr Deborah A. Lewinsohn

This file contains all reviewer reports in order by version, followed by all author rebuttals in order by version.

Version 0:

Reviewer comments:

Reviewer #1

(Remarks to the Author)
Kain et al. present a comprehensive characterization of the human neonatal MR1-restricted T (MR1T) cell repertoire in comparison to that of adults. By sorting MR1-5-OP-RU tetramer-positive T cells from cord blood (CB) and adult peripheral blood (PB) donors (n=5 each), the authors delineate distinct profiles using scRNA-seq, phenotypic analyses, and TCR sequencing. In adults, MR1T cells were predominantly canonical MAIT cells expressing the TRAV1-2 chain, with restricted TRBV usage, and displaying a $CD8^+$, $CD26^+$, $CD161^+$ phenotype. In contrast, CB-derived MR1T cells were largely TRAV1-2^- and exhibited unconventional features, including $CD4^+$, $CD26^-$, and $CD161^-$ expression. Functionally, neonatal MR1T cells showed reduced cytotoxic capacity and a dampened inflammatory transcriptional program, alongside a markedly more diverse TCR repertoire. Notably, CB-derived MAIT cells and TRAV1-2^- MR1T clones failed to respond to several riboflavin-producing pathogens commonly encountered in early life, such as S. aureus, S. pneumoniae, and M. tuberculosis. Structural modeling of a CB MAIT TCR-MR1-5-OP-RU complex, compared to an adult counterpart, revealed that differential β-chain interactions reduced TCR affinity for the MR1-ligand complex. These findings provide new insights into the ontogeny and function of MR1-restricted T cells and have implications for understanding early-life immunity.

Major Points
This is and interesting and well perfomed study from leaders in the MR1/MAIT cell field, particularly strong in the single-cell and TCR structural analysis. However, some questions remain regarding MR1 T cell repertoire ontogeny and the less developed functional and cellular aspects:
1. Comparison of ex vivo vs. expanded MR1T cells:
It would be informative to compare the phenotype, functional responses, and TCR usage of MR1/5-OP-RU tet^+ cells enriched directly ex vivo from CB and adult PBMCs with those that expand in vitro following (potentially repeated) stimulation with 5-OP-RU. Such experiments might reveal whether antigen stimulation promotes outgrowth of more responsive, possibly TRAV1-2^+, MR1-restricted T cells in CB samples.
2. Comparison to thymic MR1T cell signatures:
The authors should compare their CB-derived MR1T cell data with the recently published TCR-seq and transcriptional profiles of human thymic MR1/5-OP-RU tet^+ cells by Loh et al. (https://doi.org/10.1016/j.celrep.2024.114705). Identifying overlaps or distinctions could help elucidate developmental trajectories and contextualize the differences observed between neonatal and adult peripheral MR1T cells.

(Remarks on code availability)

Reviewer #2

(Remarks to the Author)
This manuscript by Kain et al. presents a compelling and comprehensive analysis of MR1T cells in cord blood (CB) compared to adults, offering valuable insight into early-life immune development and its contribution to neonatal infection susceptibility. The authors employed a wide range of approaches to demonstrate that CB-derived MR1T cells exhibit distinct

TCR usage and transcriptional profiles, with notable differences between unstimulated and stimulated conditions. They further cloned soluble extracellular domains of MAIT TCRs from both adult and CB sources, using SPR and crystallography to reveal structural features underlying the reduced ligand-binding affinity of neonatal TCRs. Notably, despite their diverse TCR repertoires and reduced canonical TRAV1-2/TRAJ33 usage, CB MR1T cells showed limited responsiveness to common microbial pathogens, though some retained the capacity for pro-inflammatory responses. While TRAV1-2⁻ MR1T cells were previously thought to be self-reactive, the study also finds that CB-derived clones can recognize microbes such as E. coli but not self-antigens. Together, these findings underscore the functional immaturity and broad developmental potential of neonatal MR1T cells and suggest that environmental exposures after birth are key to shaping their effector function.

Overall, I thoroughly enjoyed reading the manuscript. The authors have beautifully and convincingly described an intriguing, age-dependent development of a unique and functionally important T cell subset. The experiments are thoughtfully designed, and many well-executed controls effectively rule out alternative interpretations. I have only a few suggestions that I hope can further strengthen the manuscript:

1. I was slightly unclear on the developmental timeline of the TRAV1-2⁻ MR1T cells identified in cord blood. The study highlights their presence in CB but not in adult PBMCs, and suggests a potential for microbe-driven expansion during the first year of life. However, since CB-derived cells likely originate during the fetal period, it remains a bit uncertain for me whether similar TRAV1-2⁻ MR1T populations are also dominant in newborn or infant samples. Clarification on this point would help frame their developmental trajectory more clearly.

2. Do the authors speculate whether this is driven by differential regulation during fetal versus newborn/infant thymic selection? Additionally, do the authors expect similar developmental patterns in murine models?

3. It would be helpful if the authors could discuss whether these CB-derived TRAV1-2⁻ MR1T cells persist into adulthood, and if so, whether they retain similar phenotypic or functional characteristics. From Supplementary Fig. 1, there appears to be a detectable population of CD4⁺ TRAV1-2⁻ MR1T cells in adult samples. Would a comparison between CB- and adult-derived TRAV1-2⁻ MR1T cells be feasible?

(Remarks on code availability)

Reviewer #3

(Remarks to the Author)
In this comprehensive manuscript, Kain et al. characterized human neonatal MR1T cells isolated from cord blood (CB). Compared to MR1T cells from adults, CB MR1T cells showed greater diversity in TCR gene usage and a less cytotoxic and pro-inflammatory phenotype. In addition, in what is the most interesting finding of this study, CB-derived MR1T cell clones failed to recognize several riboflavin-producing childhood pathogens. It should be noted, however, that only four clones (A1, A2, A4) were tested, and that there could be others more closely resembling adult-derived clones.
The authors measured the affinity of one CB MAIT TCR (A2) for MR1-5-OP-RU by SPR and found it to be ~5-fold lower than the affinities of adult MAIT TCRs A-F7 and #6. Whether lower affinity is characteristic of CB MAIT TCRs is not addressed. At minimum, the authors should also determine affinities for TCRs A1 and A4.
The crystal structure of CB MAIT TCR A2 bound to MR1-5-OP-RU is very similar to previous structures of adult MAIT TCRs insofar as docking topology and specific interactions of TRAV1-2 with MR1 are concerned. Does the new structure provide any insights into the lack of reactivity of CB-derived clones towards riboflavin-producing pathogens S. aureus, S. pneumonia, or M. tuberculosis?

(Remarks on code availability)

Version 1:

Reviewer comments:

Reviewer #1

(Remarks to the Author)
The authors have thoroughly revised the study and addressed all my queries. I am happy with the new version of the manuscript.

Reviewer #2

(Remarks to the Author)
Beautiful paper. I thoroughly enjoyed the revised manuscript. Congratulations to the team on such great work!

Reviewer #3

(Remarks to the Author)
The authors have responded satisfactorily to the previous critiques.

Open Access This Peer Review File is licensed under a Creative Commons Attribution 4.0 International License, which permits use, sharing, adaptation, distribution and reproduction in any medium or format, as long as you give appropriate credit to the original author(s) and the source, provide a link to the Creative Commons license, and indicate if changes were made.
In cases where reviewers are anonymous, credit should be given to 'Anonymous Referee' and the source.
The images or other third party material in this Peer Review File are included in the article's Creative Commons license, unless indicated otherwise in a credit line to the material. If material is not included in the article's Creative Commons license and your intended use is not permitted by statutory regulation or exceeds the permitted use, you will need to obtain permission directly from the copyright holder.
To view a copy of this license, visit https://creativecommons.org/licenses/by/4.0/

We appreciate the reviewers' constructive comments, which were of considerable help in formulating a greatly improved manuscript. Below, we have attempted to provide a point-by-point response to each reviewer comment, including a description of any newly added data to the manuscript. In the following, we have used text in italics to reproduce the reviewers' comments verbatim.

<u>Reviewer #1</u>

Kain et al. present a comprehensive characterization of the human neonatal MR1-restricted T (MR1T) cell repertoire in comparison to that of adults. By sorting MR1-5-OP-RU tetramer positive T cells from cord blood (CB) and adult peripheral blood (PB) donors (n=5 each), the authors delineate distinct profiles using scRNA-seq, phenotypic analyses, and TCR sequencing. In adults, MR1T cells were predominantly canonical MAIT cells expressing the TRAV1-2 chain, with restricted TRBV usage, and displaying a CD8^{+}, CD26^{+}, CD161^{+} phenotype. In contrast, CB-derived MR1T cells were largely TRAV1-2^{-} and exhibited unconventional features, including CD4^{+}, CD26^{-}, and CD161^{-} expression. Functionally, neonatal MR1T cells showed reduced cytotoxic capacity and a dampened inflammatory transcriptional program, alongside a markedly more diverse TCR repertoire. Notably, CB-derived MAIT cells and TRAV1-2^{-} MR1T clones failed to respond to several riboflavin-producing pathogens commonly encountered in early life, such as S. aureus*,* S. pneumoniae*, and* M. tuberculosis*. Structural modeling of a CB MAIT TCR-MR1-5-OP-RU complex, compared to an adult counterpart, revealed that 7 of 9 differential β-chain interactions reduced TCR affinity for the MR1-ligand complex. These findings provide new insights into the ontogeny and function of MR1-restricted T cells and have implications for understanding early-life immunity.*

Major Points

This is an interesting and well performed study from leaders in the MR1/MAIT cell field, particularly strong in the single-cell and TCR structural analysis. However, some questions remain regarding MR1 T cell repertoire ontogeny and the less developed functional and cellular aspects:

Thank you for your careful review of our manuscript. We have attempted to address your questions regarding MR1T cell repertoire ontogeny and functional and cellular aspects of MR1T cells in the responses below.

1. Comparison of ex vivo vs. expanded MR1T cells:

It would be informative to compare the phenotype, functional responses, and TCR usage of MR1/5-OP-RU tet^{+} cells enriched directly ex vivo from CB and adult PBMCs with those that expand in vitro following (potentially repeated) stimulation with 5-OP-RU. Such experiments might reveal whether antigen stimulation promotes outgrowth of more responsive, possibly TRAV1-2^{+}, MR1-restricted T cells in CB samples.

<u>Response:</u> We appreciate this feedback and opportunity to strengthen our findings. We have now completed this experiment and have included the results in the revised manuscript (Figure 4). Briefly, we found that stimulating MR1T cells with 5-OP-RU in vitro resulted in a dramatic and selective expansion of TRAV1-2(+) MAIT cells as compared to TRAV1-2(-) MR1T cells in neonatal CBMC (*n*=3). These in vitro findings are consistent with our hypothesis that post-natal exposure to 5-OP-RU, presumably as produced by commensal bacteria, preferentially expands TRAV1-2(+) MAIT cells. This figure and figure legend is reproduced below.

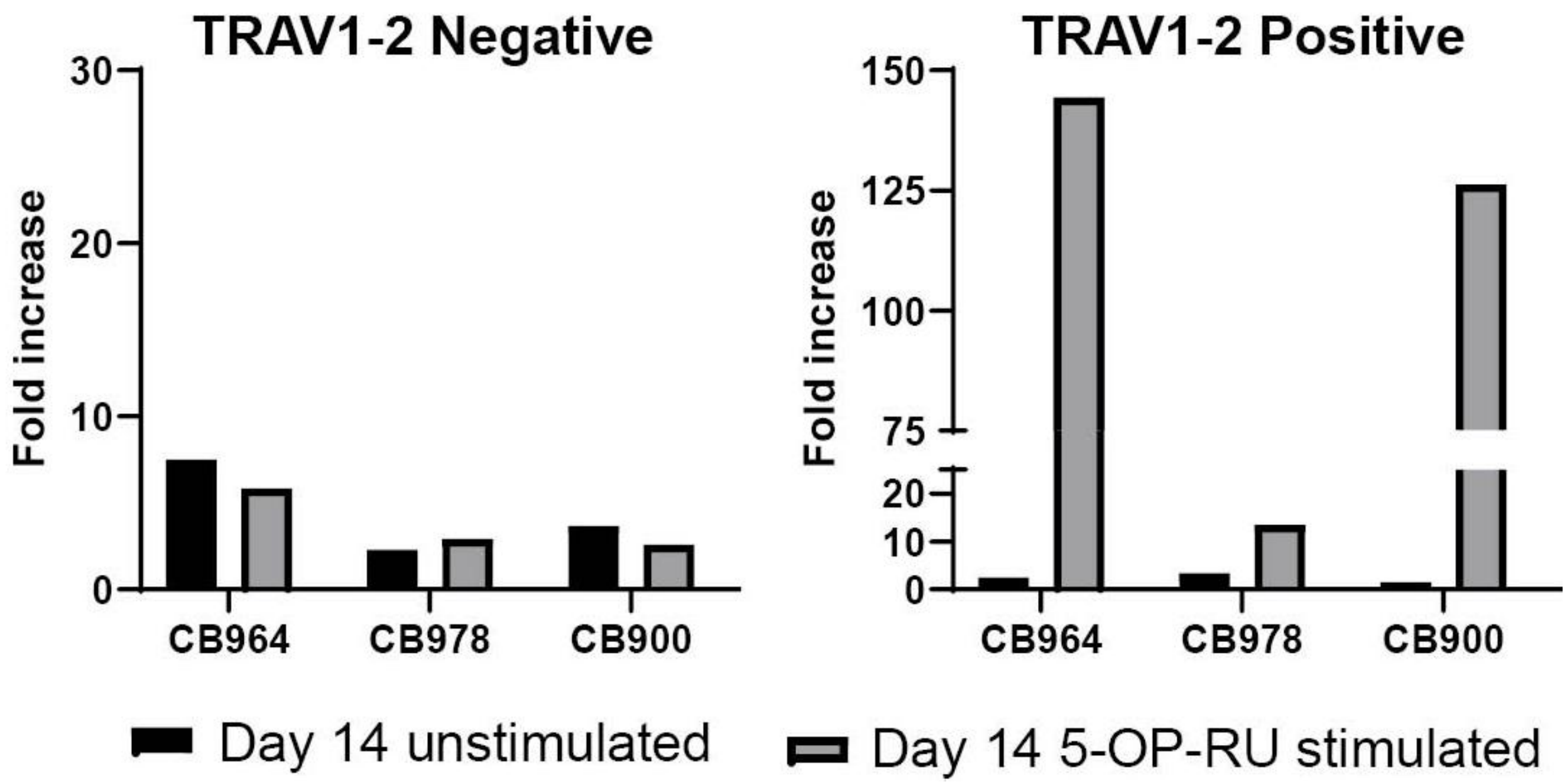

Figure 4 (new): 5-OP-RU preferentially expands MAIT cells compared to TRAV1-2(-) MR1T cells

A-B) CBMC (*n*=3) had phenotyping performed by flow cytometry on day 0. The remaining cells were either stimulated on day 1 and day 7 with 5-OP-RU (1 µM) or unstimulated. Cells were harvested and analyzed by flow cytometry on Day 14. Cells were gated on live, CD3+ MR1/5-OP-RU+ and either TRAV1-2+ or TRAV1-2-. The fold increase on day 14 compared to the day 0 frequencies is shown for both the unstimulated and 5-OP-RU stimulated MR1T cells.

2. Comparison to thymic MR1T cell signatures:
The authors should compare their CB-derived MR1T cell data with the recently published TCR-seq and transcriptional profiles of human thymic MR1/5-OP-RU tet⁺ cells by Loh et al. (https://doi.org/10.1016/j.celrep.2024.114705). Identifying overlaps or distinctions could help elucidate developmental trajectories and contextualize the differences observed between neonatal and adult peripheral MR1T cells.

Response: We thank the reviewer for highlighting the recent work by Loh et al. (*Cell Reports*, 2024; https://doi.org/10.1016/j.celrep.2024.114705), which provides an in-depth single-cell RNA-seq and TCR-seq analysis of human thymic TRAV1-2(+) MR1/5-OP-RU tet⁺ cells. We agree that this dataset represents a valuable resource for understanding developmental trajectories of MR1T cells. We have now reviewed the transcriptional profiles and TCR usage data made publicly available under GEO accession GSE249684 and compared to our dataset. Specifically, as the dataset from Loh et al. included only TRAV1-2(+) MAIT cells, we compared our data from TRAV1-2(+) MAIT cells to this publicly available dataset.

Unsupervised clustering of the CB MAIT cells from our data set, thymic MAIT cells from the Loh et al. data set, and adult MAIT cells from both data sets demonstrates spatially distinct clusters (Response Figure 1). Cord blood and thymic MAIT cells cluster separately, supporting differential expression profiles between these two cell populations. However, comparison of adult MAIT cells from our study and to those of Loh et al., which should represent very similar MAIT cell populations, also cluster separately (Response Figure 1A). This likely demonstrates a

significant batch effect between these two datasets reflecting technical and experimental variation arising from data generated under distinct experimental conditions and performed in different laboratories. This suggests critical limitations in our ability to draw robust conclusions from this analysis when comparing the data derived from the separate studies. Given this limitation, we have elected to not perform any additional analysis comparing data between studies.

However, we did apply our gene module scores we utilized in our manuscript to the Loh et al data, and we found that the major themes of our paper also hold true. Specifically, similar to the enrichment in CB MAIT cells, the naïve T cell score was enriched in thymus MAIT cells (Response Figure 1A-C). In contrast the cytotoxicity score was enriched in adult MAIT cells in both datasets (Response Figure 1 D-F). We have added this reference to the discussion in our revised manuscript and added some additional context to the discussion as shown:

"These findings are similar to recently published work from Loh et al. comparing MAIT cells from thymic samples to adult PBMC MAIT cells[43]. They similarly found that thymic MAIT cells had upregulation of naïve T cell genes compared to adult MAIT cells. By contrast, the cytotoxicity score was upregulated in adult MAIT cells as compared to thymic MAIT cells."

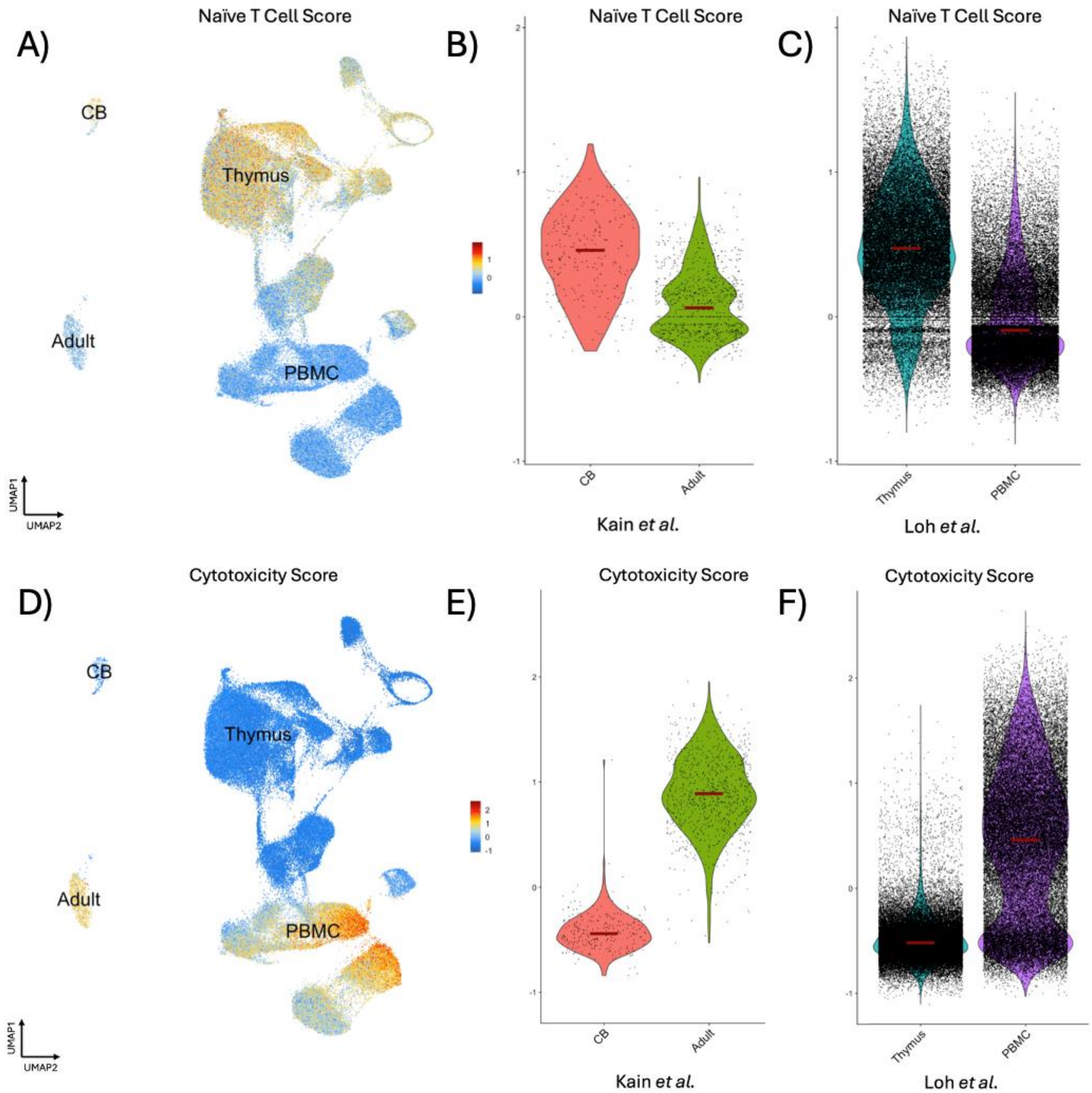

Response Figure 1: A) UMAP of CB and adult TRAV1-2(+) MAIT cells from Kain et al. combined with thymus and adult MAIT cells from Loh et al. Naïve T cell score (CTSH, CA6, GSTT1, LEF1, RGS10, TMIGD2) is overlaid over the UMAP. B) Violin plot is shown quantifying the naïve T cell score for CB as compared to adult MAIT cells in our dataset. C) Violin plot is shown quantifying the naïve T cell score for thymus and adult PBMC MAIT cells from Loh et al. D) UMAP of CB and adult TRAV1-2(+) MAIT cells from Kain et al. combined with thymus and adult MAIT cells from Loh et al. Cytotoxicity score (PRF1, GNLY, NKG7, GZMA, GZMB, GZMH, GZMK, GZMM) is overlaid over the UMAP. E) Violin plot is shown quantifying the cytotoxicity score for CB compared to adult MAIT cells in our data set. F) Violin plot is shown quantifying the cytotoxicity score for thymus and adult PBMC MAIT cells from Loh et al.

Reviewer #2

This manuscript by Kain et al. presents a compelling and comprehensive analysis of MR1T cells in cord blood (CB) compared to adults, offering valuable insight into early-life immune development and its contribution to neonatal infection susceptibility. The authors employed a wide range of approaches to demonstrate that CB-derived MR1T cells exhibit distinct TCR usage and transcriptional profiles, with notable differences between unstimulated and stimulated conditions. They further cloned soluble extracellular domains of MAIT TCRs from both adult and CB sources, using SPR and crystallography to reveal structural features underlying thereduced ligand-binding affinity of neonatal TCRs. Notably, despite their diverse TCR repertoires and reduced canonical TRAV1-2/TRAJ33 usage, CB MR1T cells showed limited responsiveness to common microbial pathogens, though some retained the capacity for pro-inflammatory responses. While TRAV1-2⁻MR1T cells were previously thought to be self-reactive, the study also finds that CB-derived clones can recognize microbes such as E. coli *but not self-antigens. Together, these findings underscore the functional immaturity and broad developmental potential of neonatal MR1T cells and suggest that environmental exposures after birth are key to shaping their effector function.*

Overall, I thoroughly enjoyed reading the manuscript. The authors have beautifully and convincingly described an intriguing, age-dependent development of a unique and functionally important T cell subset. The experiments are thoughtfully designed, and many well-executed controls effectively rule out alternative interpretations. I have only a few suggestions that I hope can further strengthen the manuscript:

Your suggestions, which are addressed in detail below, are greatly appreciated.

1.I was slightly unclear on the developmental timeline of the TRAV1-2⁻MR1T cells identified in cord blood. The study highlights their presence in CB but not in adult PBMCs and suggests a potential for microbe-driven expansion during the first year of life. However, since CB-derived cells likely originate during the fetal period, it remains a bit uncertain for me whether similar TRAV1- MR1T populations are also dominant in newborn or infant samples. Clarification on this point would help frame their developmental trajectory more clearly.

Response: Since we do not have infant samples in this study, we cannot directly compare to cord blood samples here, but we have explored this previously (doi: 10.3389/fimmu.2020.556695). In that study we found that by 10 weeks of age, TRAV1-2(-) MR1T cells had fallen to a similar proportion as in adults. Furthermore, in CB donors there was a significant increase in the proportion of TNF producing MR1T cells (in response to both M smeg and PMA/Ionomycin) in TRAV1-2(+) compared to TRAV1-2(-) cells, but this increased further in infant donors. We have also further explored this in more depth in a recent pre-print (doi**:** https://doi.org/10.1101/2025.10.09.681353) where single cell sequencing was performed on CB, infant and adult MR1T cells. Here we found that TRAV1-2(-) MR1T cells at 10 weeks of age, retain a naïve T cell gene profile, which is similar to CB MR1T cells. By contrast, infant TRAV1-2(+) MR1T cells demonstrate a diminished naïve T cell gene profile. By adulthood, however, TRAV1-2(-) MR1T cells have lost this high naïve T cell gene signature, suggesting their maturation occurs, but at a slower timeframe compared to TRAV1-2(+) MR1T cells.

Additional context is added to the discussion as below:
"We have also recently explored this development in more detail, looking at the specific development of MAIT cells compared to TRAV1-2(-) MR1T cells in CB, 10-week-old and adult participants[41]. In that work, we found that by 10-weeks MAIT cells had upregulated cytotoxic genes and downregulated naïve genes, whereas this shift was not seen until adulthood for TRAV1-2(-) MR1T cells, suggesting they maturation happens after 10 weeks of age. In addition, MAIT cells rapidly expand in early life, whereas TRAV1-2(-) MR1T cells persist as a relatively fixed proportion of all T cells in adult populations."

2. Do the authors speculate whether this is driven by differential regulation during fetal versus newborn/infant thymic selection? Additionally, do the authors expect similar developmental patterns in murine models?

Response: As transcriptional profiling of human thymus specimens was not included in this study and, to our knowledge, literature describing transcriptional profiling of human thymus is limited to the Loh et al. study above, which didn't study fetal thymus, we hesitate to speculate regarding differential regulation of thymic selection in the fetus as compared to neonates/infants. Rather, we speculate that these current data as well as our prior work (doi: 10.3389/fimmu.2020.556695 and doi**:** https://doi.org/10.1101/2025.10.09.681353) support that postnatal selective expansion of TRAV1-2+ MR1T cells as compared to TRAV1-2- MR1T cells is due to exposure to 5-OP-RU producing microorganisms.

Regarding murine models, the publications by Constantinides et al. (doi: 10.1126/science.aax6624) and Legoux et al. (doi: 10.1126/science.aaw2719) are highly relevant and support similar postnatal trajectories of MR1T cells. Constantinides et al. showed that the expansion and establishment of mucosal colonization of MAIT cells (as defined by 5-OP-RU-tetramer staining) is dependent upon postnatal intestinal colonization with 5-OP-RU-producing microorganisms. Legoux et al. demonstrated similar findings and in addition show that 5-OP-RU is getting into the thymus and selecting for MAIT cells. We appreciate the suggestion to consider evidence from murine models and have added discussion of these papers to the revised manuscript.

Additional context is added to the discussion as below:
"The selective expansion of MAIT cells we found following 5-OP-RU stimulation, suggests that exposure to 5-OP-RU following birth could represent a driving force for this shift from predominantly TRAV1-2(-) MR1T cells to predominantly MAIT cells later in life. This is supported in murine models showing that establishment of mucosal colonization of MAIT cells is dependent on intestinal colonization of 5-OP-RU producing microorganisms[21,46]."

3. It would be helpful if the authors could discuss whether these CB-derived TRAV1-2$^-$ MR1T cells persist into adulthood, and if so, whether they retain similar phenotypic or functional characteristics. From Supplementary Fig. 1, there appears to be a detectable population of CD4$^+$ TRAV1-2$^-$ MR1T cells in adult samples. Would a comparison between CB- and adult-derived TRAV1-2$^-$ MR1T cells be feasible?

Response: Regarding persistence of TRAV1-2(-) MR1T cells into adulthood, Figure 2D shows the proportion of MR1T cells in adults expressing TRAV 1-2 transcripts, revealing that while the majority of adult MR1T cells express TRAV1-2, a minority of adult MR1T cells express non-TRAV1-2 TCR transcripts. In addition, as Reviewer 2 points out, Supplemental Figure 1C&D demonstrate a small population of CD4$^+$ TRAV1-2(-) MR1T cells that persist in adults. This

small population is more clearly demonstrated in Figure 3B of our prior publication (doi: 10.3389/fimmu.2020.556695) in which we used TRAV1-2 and CD4 antibodies with analysis by flow cytometry to demonstrate persistence of this population in adolescents (shown in a reproduction of Figure 3B&D below, Response Figure 2) and adults.

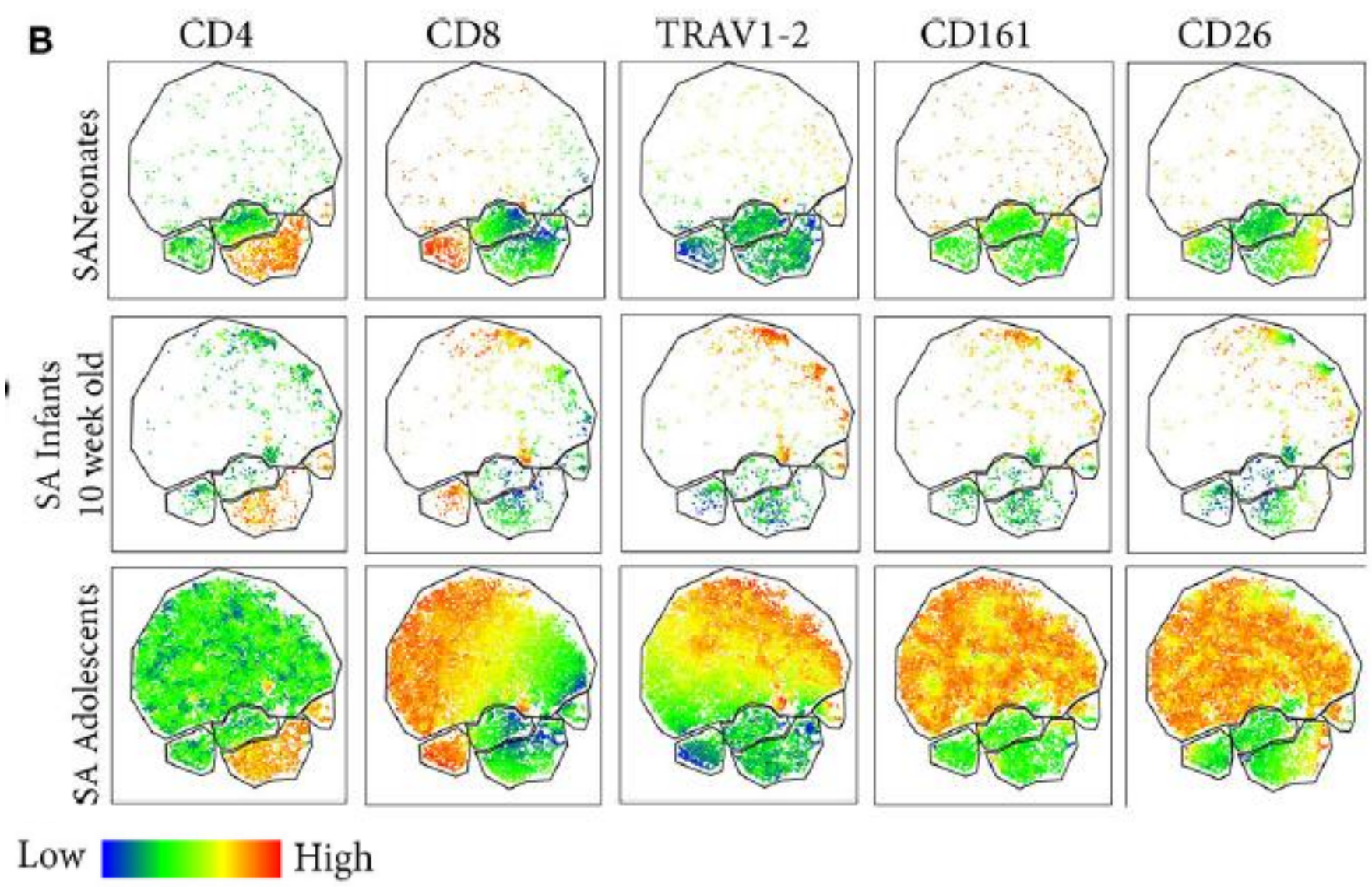

Response Figure 2: Reprint of Figure 3B from (doi: 10.3389/fimmu.2020.556695). Phenotypic diversity of MR1T cells in individuals of different ages. t-Distributed stochastic neighbor embedding (tSNE) plots showing MR1-R-OP-RU tetramer+CD3+ T cells from South Africa (SA). Plots represent cells from 10 samples from each age group. Dot plots colored by the relative expression level of each marker (CD4, CD8, TRAV1-2, CD161, or CD26) in tetramer-defined MR1T cells for each age group in SA. Red= highest expression, blue= lowest expression.

We have clarified the discussion of our prior published results in the introduction of the revised manuscript as follows:
"TRAV1-2(-) MR1T cells represent the majority of MR1T cells in cord blood (CB) samples[21]. By contrast, while a TRAV1-2(-) MR1T cell population persists in adults, MAIT cells represent the vast majority of MR1T cells."

We have clarified findings of this current study in the revised manuscript as follows:
"Conversely, while the vast majority of adult MR1T cells express TRAV1-2, a population of MR1T cells expressing other TRAV genes are present in adults (Figure 2D)."

Furthermore, we speculate that the TRAV1-2(-) MR1T cells that persist into adulthood, are not unlike naïve conventional T cells that persist into adulthood, in that these cells have not yet experienced activating ligands. For example, our previous work demonstrates that a TRAV-12(+) MR1T cell clone isolated from an adult recognizes *S. pyogenes*, which doesn't produce 5-OP-RU, and poorly recognizes riboflavin producing organisms. These data and that several alternative MR1T ligands have been described by our group support this assertion.

We have added a more detailed discussion of these points in the revised manuscript as follows:

"Conversely, we believe that the TRAV1-2(-) MR1T cells persist in adults at frequencies comparable to other T cell subsets. Furthermore, we speculate that the TRAV1-2(-) MR1T cells that persist into adulthood, are not unlike naïve conventional T cells that persist into adulthood, in that these cells have not yet experienced activating ligands. For example, our previous work demonstrating that a TRAV-12(+) MR1T cell clone isolated from an adult recognizes *S. pyogenes*, which doesn't produce 5-OP-RU, and poorly recognizes riboflavin producing organisms[6]. This is further supported by work from Harriff et al. demonstrating an unexpectedly diverse MR1 ligandome[5]."

Differential gene expression between adult and CB-derived TRAV1-2(-) MR1T cells, demonstrates that adult TRAV1-2(-) MR1T cells had upregulation of many genes including many cytotoxic and effector genes (CCL4, CCL5, GZMA, GZMH, KLRBA, CST7 etc.), whereas CB TRAV1-2(-) MR1T cells had upregulation of many naïve associated genes (CCR7, SELL, TCF7; Response Figure 3). We have elected to not include this in the manuscript itself as we have recently published a pre-print (doi**:** https://doi.org/10.1101/2025.10.09.681353) where this is further explored in more detail, finding that TRAV1-2(-) MR1T cells appear to mature more slowly than MAIT cells. In this manuscript we show that 10 week-old infant TRAV1-2(-) MR1T cells express high naïve T cell gene signatures similar to neonatal TRAV1-2(-) MR1T cells, while 10 week-old infant MAIT cells appear more similar to adult MAIT cells (Fig 3A,B of doi**:** https://doi.org/10.1101/2025.10.09.681353).

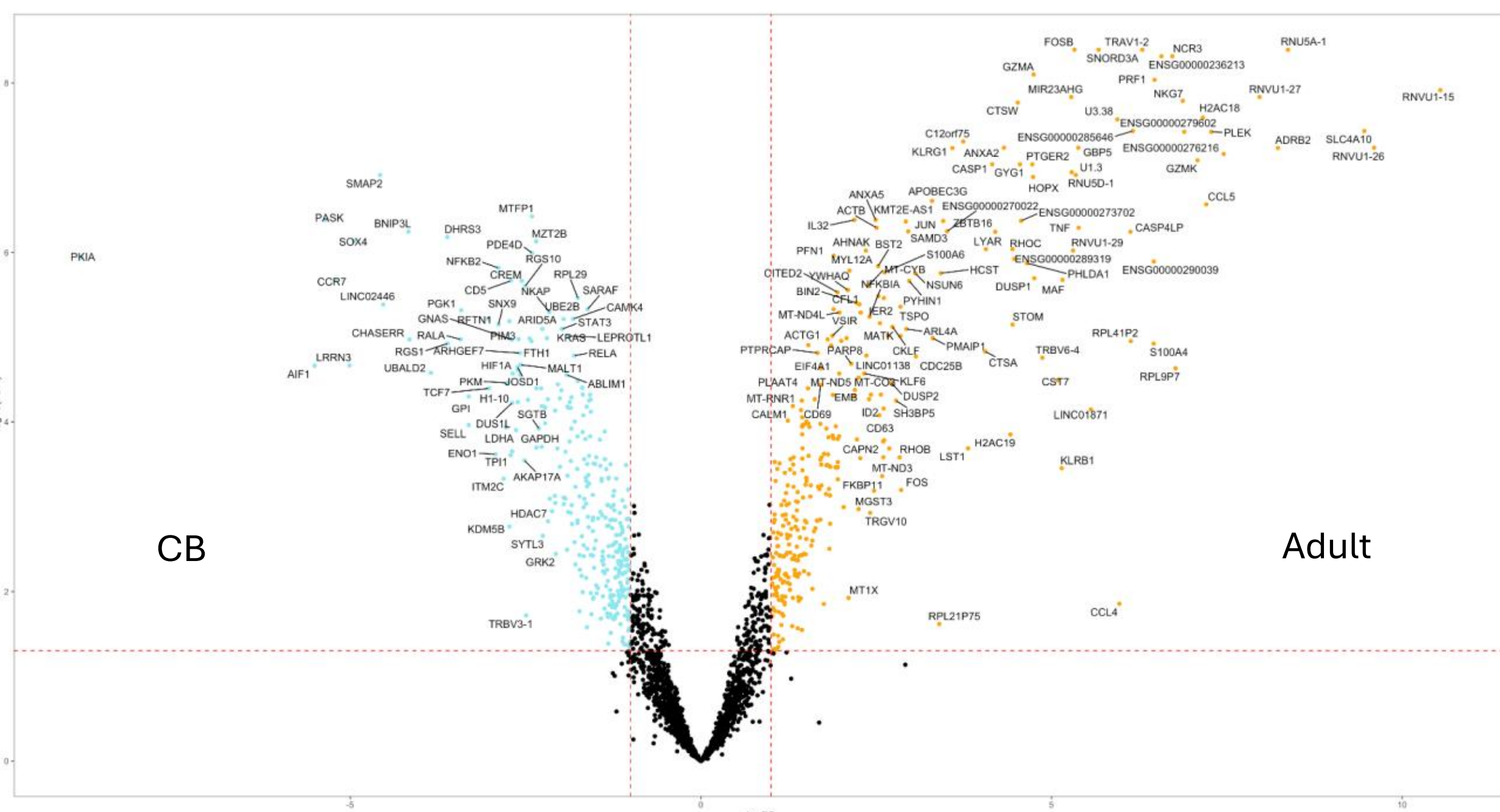

Response Figure 3: Volcano plot for differential gene expression of CB TRAV1-2(-) MR1T cells vs Adult TRAV1-2(-) MR1T cells. Labeled genes were greater than 0.5 or less than -0.5 log fold change and had a Bonferroni-adjusted p-value of < 0.05.

<u>Reviewer #3</u>

In this comprehensive manuscript, Kain et al. characterized human neonatal MR1T cells isolated from cord blood (CB). Compared to MR1T cells from adults, CB MR1T cells showed greater

diversity in TCR gene usage and a less cytotoxic and pro-inflammatory phenotype. In addition, in what is the most interesting finding of this study, CB-derived MR1T cell clones failed to recognize several riboflavin-producing childhood pathogens. It should be noted, however, that only four clones (A1, A2, A4) were tested, and that there could be others more closely resembling adult-derived clones.

<u>Response:</u> We agree. While Figure 6B supports that the three clones tested are largely representative of this neonate's MR1T repertoire, we acknowledge that our study of T cell clones from just one cord blood specimen limits the generalizability of our results. We have clarified this point in the discussion.

Specifically, we add the following text to the discussion:
"To study the pathogen recognition pattern and functional capacity of CB MR1T cells, we studied three MR1T cell clones isolated from CB of one individual. While we acknowledge that these T cell clones likely do not fully represent either this individual's MR1T cell population nor neonatal MR1T cell populations in general, these T cell clones demonstrated similar diversity of CD4/CD8 and TCR expression to this neonate's ex vivo MR1T cell population as examined by single cell RNA sequencing. Moreover, the gene expression profile of the three unstimulated cord blood clones was similar to that observed in the ex vivo MR1T cell data derived from five CB specimens."

"While the generalizability of these results from three CB MR1T cell clones from one individual to neonatal MR1T cells in general is limited, we speculate that the diminished response of neonatal MR1T cells to common childhood pathogens, could reflect a hole in the repertoire of the MR1T cells' response to these organisms at birth and that this defect is repaired by environmental exposures after birth."

The authors measured the affinity of one CB MAIT TCR (A2) for MR1-5-OP-RU by SPR and found it to be ~5-fold lower than the affinities of adult MAIT TCRs A-F7 and #6. Whether lower affinity is characteristic of CB MAIT TCRs is not addressed. At minimum, the authors should also determine affinities for TCRs A1 and A4.

<u>Response:</u> We have now measured the binding affinities of two additional CB TCRs (A1 and A4) toward MR1-5-OP-RU (revised Figure 9) and notably the three CB MR1T TCRs showed similar modest affinities toward this MR1-antigen. This updated figure is shown below:

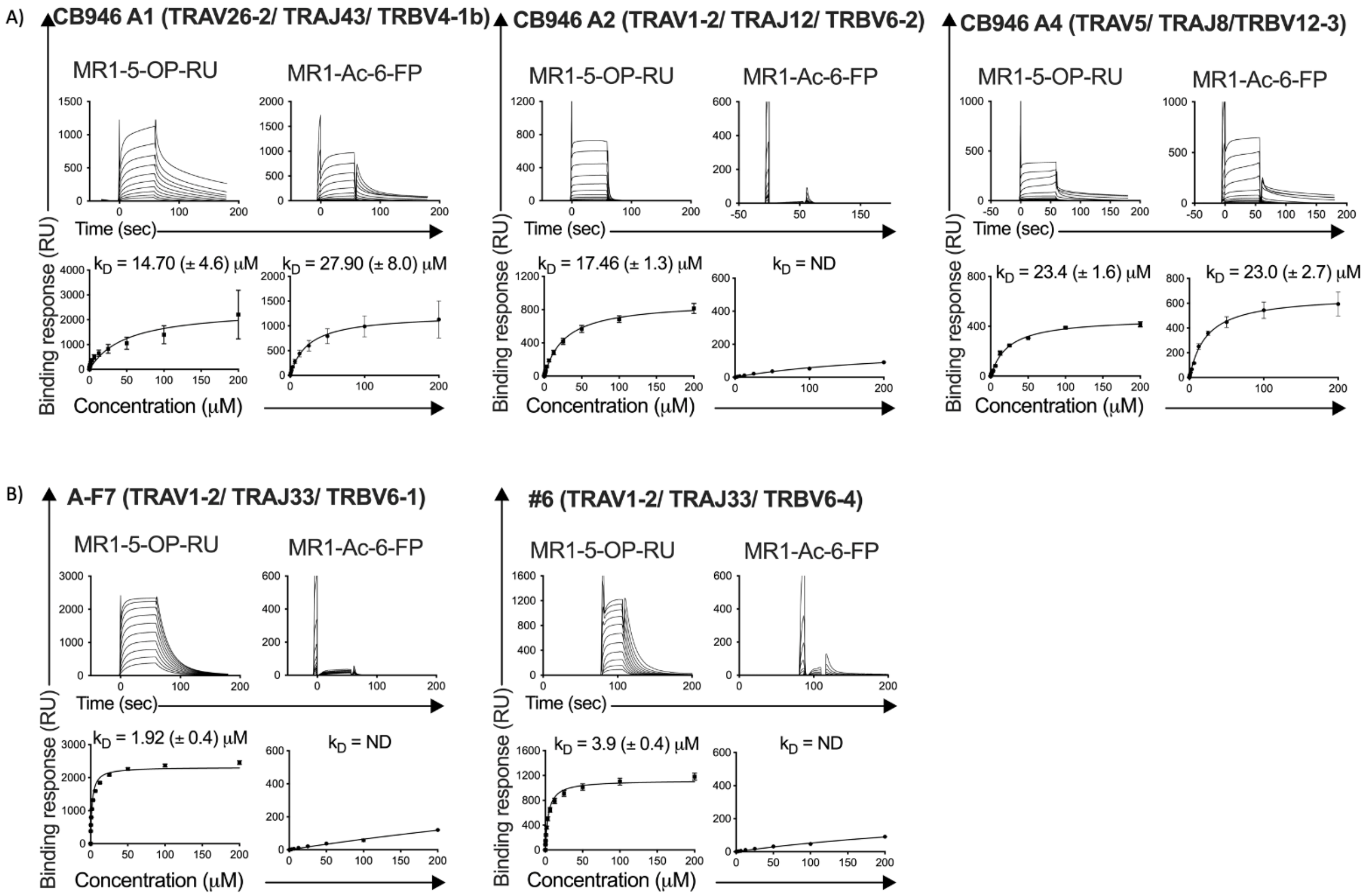

Figure 9 (revised): Steady-state affinity measurements of soluble TCRs for MR1-antigen complexes

a) TCR αβ gene names and CDR amino acid sequences of the TCR cell clones.

b) The affinity of TCR-MR1-Ag interactions were determined using SPR, by measuring the binding of various concentrations of soluble adult MAIT TCRs of A-F7 (TRAV1-2/TRBV6-1; left panel) and #6 (TRAV1-2/TRBV6-4; middle panel), as well as cord CB946 A2 TCR clone (TRAV1-2/TRBV6-2; right panel) against human MR1 refolded with 5-OP-RU and Ac-6-FP antigens. The SPR runs were conducted as duplicate in two independent experiments using different batches of proteins. Experiments were conducted with serial dilutions of the TCRs. The SPR sensograms (upper panels), equilibrium curves (lower panels) and steady state KD values (μM) were prepared in GraphPad Prism 10. Error bars represent the mean and SD from technical replicates (two independent experiments). ND, not determined. RU, response unit

The crystal structure of CB MAIT TCR A2 bound to MR1-5-OP-RU is very similar to previous structures of adult MAIT TCRs insofar as docking topology and specific interactions of TRAV1-2 with MR1 are concerned. Does the new structure provide any insights into the lack of reactivity of CB-derived clones towards riboflavin-producing pathogens S. aureus, S. pneumoniae, *or* M. tuberculosis*?*

__Response:__ We speculate that while the β-chain contacts may contribute to the lack of responsiveness to these microbes, there are likely several additional contributing factors. In the revised manuscript we have clarified our discussion of contributing factors and expanded the discussion to include our recently published manuscript describing that host riboflavin catabolites inhibit MAIT cell activation to pathogen-derived ligands.

“Despite all CB-derived clones staining for MR1/5-OP-RU-tetramer, none of these clones responded to some common riboflavin-producing childhood pathogens, *S. aureus, S. pneumoniae and M. tuberculosis*. The reasons for this discrepancy are not entirely clear, but there are several possible explanations. As among MAIT cells, the TCR β-chain can contribute to MR1 ligand discrimination [37,48,49], one possibility is that the less typical β-chain contacts for the CB MAIT cell clone TCR contributes to the lack of reactivity towards these riboflavin-producing pathogens. Another possibility is that the concentration of ligand processed and presented during the infection with these pathogens could be insufficient to activate these clones. Supporting this, we see that one of the CB T cell clones responds to PL1, which can be found in *M. smegmatis* supernatants[5]. However, we do not know the extent to which it is produced by live *M. smegmatis* and hence the quantity of this ligand may well be insufficient to activate our T cell clone. In addition, we have recently shown that host cell riboflavin and its catabolites can bind to MR1, reducing MR1 expression at the cell surface and inhibiting MAIT cell activation by pathogen-derived MR1 ligands [5,50]. Another possibility is that the optimal ligands for the TCRs expressed by CB-derived MR1T cell clones are not produced by these organisms. In this regard for adults, 5-amino-6-D-ribityaminouracil (5-A-RU) is a key precursor for the production of MAIT cell ligands[51], but this may not be the case for the diverse TCRs expressed by CB MR1T cells. It is therefore possible that these CB TCRs could recognize 5-OP-RU, but not a sufficiently activating ligand produced by these particular riboflavin-producing organisms.”

Reviewer comments to the revised submission and our responses are listed below.

Reviewer #1
The authors have thoroughly revised the study and addressed all my queries. I am happy with the new version of the manuscript.

Response: Thank you for reviewing the revised manuscript. We are glad that we were able to satisfactorily address all your queries.

Reviewer #2
Beautiful paper. I thoroughly enjoyed the revised manuscript. Congratulations to the team on such great work!

Response: Thank you so much!

Reviewer #3
The authors have responded satisfactorily to the previous critiques.

Response: Thank you for reviewing the revised manuscript. We are glad that we were able to satisfactorily address your previous critiques.
